# Recommendations of scRNA-seq Differential Gene Expression Analysis Based on Comprehensive Benchmarking

**DOI:** 10.3390/life12060850

**Published:** 2022-06-07

**Authors:** Jake Gagnon, Lira Pi, Matthew Ryals, Qingwen Wan, Wenxing Hu, Zhengyu Ouyang, Baohong Zhang, Kejie Li

**Affiliations:** 1 Analytics and Data Sciences, Biogen, Inc., 225 Binney St., Cambridge, MA 02142, USA; jake.gagnon@biogen.com; 2 PharmaLex, 1700 District Ave., Burlington, MA 01803, USA; lira.pi@pharmalex.com (L.P.); matthew.ryals@pharmalex.com (M.R.); qingwen.wan@pharmalex.com (Q.W.); 3 Research Department, Biogen, Inc., 225 Binney St., Cambridge, MA 02142, USA; wenxing.hu@biogen.com; 4 BioInfoRx, Inc., 510 Charmany Dr., Suite 275A, Madison, WI 53719, USA; oyoung@bioinforx.com

**Keywords:** scRNA-seq, single-cell, RNA-seq, DEG, differential expression, DE, benchmarking, scRNA-seq simulator

## Abstract

To guide analysts to select the right tool and parameters in differential gene expression analyses of single-cell RNA sequencing (scRNA-seq) data, we developed a novel simulator that recapitulates the data characteristics of real scRNA-seq datasets while accounting for all the relevant sources of variation in a multi-subject, multi-condition scRNA-seq experiment: the cell-to-cell variation within a subject, the variation across subjects, the variability across cell types, the mean/variance relationship of gene expression across genes, library size effects, group effects, and covariate effects. By applying it to benchmark 12 differential gene expression analysis methods (including cell-level and pseudo-bulk methods) on simulated multi-condition, multi-subject data of the 10x Genomics platform, we demonstrated that methods originating from the negative binomial mixed model such as glmmTMB and NEBULA-HL outperformed other methods. Utilizing NEBULA-HL in a statistical analysis pipeline for single-cell analysis will enable scientists to better understand the cell-type-specific transcriptomic response to disease or treatment effects and to discover new drug targets. Further, application to two real datasets showed the outperformance of our differential expression (DE) pipeline, with unified findings of differentially expressed genes (DEG) and a pseudo-time trajectory transcriptomic result. In the end, we made recommendations for filtering strategies of cells and genes based on simulation results to achieve optimal experimental goals.

## 1. Introduction

In recent years, single-cell RNA sequencing technology has gained popularity due to its advantages over bulk RNA sequencing [1]. This includes a better understanding of tissue heterogeneity [2], characterizations of rare cell populations [3,4], and cell type-driven disease etiology [5,6]. A key question in analyzing scRNA-seq data is the identification of cell-type specific, differentially-expressed genes between groups by using appropriate methods.

To explore this question, we need to consider the unique challenges of scRNA-seq data analysis, namely the dropout of lowly expressed genes, the sparsity of scRNA-seq datasets, and the hierarchical nature of single-cell data. To properly model these data structures, we need to consider all the relevant sources of variation in a multi-subject, multi-condition scRNA-seq experiment: the cell-to-cell variation within a subject, the variation across subjects, the variability across cell types, the mean/variance relationship of gene expression across genes, library size effects, group effects, and covariate effects.

Since the ground truth of DEGs of a real scRNA-seq dataset is often unknown, we utilized simulated datasets under various parameter settings to benchmark both bulk and single-cell DEG algorithms on multi-subject scRNA-seq datasets. In this present work, we focused on developing a novel scRNA-seq simulator that recapitulates the data characteristics of real scRNA-seq datasets, accommodates realistic designs, simulates realistic cell numbers, and accounts for all the sources of variation noted above.

Our proposed simulator focuses on the multi-sample, multi-condition scenario and improves upon previous literature with three contributions. First, we account for covariates during the simulation step (we are not aware of any other simulators in the literature that account for covariates). Second, our simulator can capture subject to subject variation, which is not modelled in previous simulators such as Splatter or scDesign. Lastly, we utilize data from all controls and all disease subjects during the estimation step of the simulator. This differs from Soneson (2018)’s work [7] which uses a single subject as the basis of estimation for their simulator.

Previous work in bulk RNA-seq/scRNA-seq simulation includes a non-parametric simulator [8], semi-parametric simulation [9], Negative Binomial simulators [10,11], and multinomial simulation [12]. Unfortunately, these simulators do not fully address the complexities of the scRNA-seq data structure. For example, SimSeq [8] uses a subsampling procedure on the columns (subjects) of pilot data and a weighted probability sampling on rows (genes) to simulate DEGs in bulk RNA-seq data. This weighted probability sampling on rows procedure does not guarantee that chosen genes are truly differentially expressed. Additionally, SimSeq does not address the single-cell RNA-seq scenario and cannot account for designs with covariates.

Addressing some of these limitations is SpSimSeq [9], a semiparametric simulator for both bulk and single-cell RNAseq datasets. This algorithm utilizes density estimation on pilot data to estimate the probability distribution of gene expression and then simulates from this distribution. Their simulator is quite flexible and can model single-cell differential expression, single-cell dropout, and the simulation of batch effects. However, the variation across subjects and covariate effects are not accommodated.

Other simulators for scRNA-seq data include Splatter [11], the ZINB model from IDEAS [11], the Negative Binomial model from Zimmerman et al. [13], and scDesign [12] which utilizes a multinomial model for differential expression. These four approaches have serious limitations: Splatter and scDesign do not simulate the multi-subject scRNA-seq scenario. It is important to note that both Splatter and scDesign can simulate the data subject by the subject using a fixed single sample as input, but this procedure does not capture subject to subject variation. The model from Zimmerman et al. will simulate data at the TPM level, but our aim is a count-level simulator, whereas IDEAS does simulate count-level data, but does not accommodate covariates during the simulation step.

The simulation approach from Squair et al. [14] is based on Splatter. As mentioned previously, Splatter does not simulate the multiple subject scRNA-seq scenario. Additionally, Squair et al. differ from this current work by not adjusting for covariates in the simulation step nor in the DEG model fitting step.

After constructing our novel scRNA-seq simulator, named MSMC-Sim (multi-subject, multi-condition simulator), we applied it to benchmark 12 differential expression (DE) methods on simulated multi-condition, multi-subject count data, namely edgeR [15], limma-voom [16], DESeq2 [17] with internal filtering and without log2FC shrinkage, DESeq2 with internal filtering and with log2FC shrinkage, DESeq2 without log2FC shrinkage and with internal filtering disabled, and four single-cell DE methods (glmmTMB [18], NEBULA-HL and NEBULA-LN [19], and MAST [20]). Previous work in the literature has addressed benchmarking pseudo bulk and single-cell DE algorithms on scRNA-seq data. Crowell et al., Miao et al., and others [7,10,14,21,22,23] compare 15 DE methods comprising both pseudo-bulk and cell-level DE methods on simulated multi-sample multi-condition scRNA-seq data. Miao et al. [21] compare 14 popular DE algorithms on single-cell mouse data, while Jaakkola [22] compare 5 DE methods on cell-level data in mouse and human tonsils.

In addition to providing a novel simulation approach, our DE benchmarking approach contains several novel features when compared to the existing DE method benchmarking literature and focuses on the multi-sample, multi-condition scenario. Our most significant contribution is that we account for covariates both in the simulation step and in the DEG model fitting step [We are not aware of any other manuscripts that benchmark while accounting for covariates]. We expect an increase in statistical power by accounting for covariate effects in the design matrix when applying DEG algorithms to simulated datasets. Additionally, our benchmarking approach is more comprehensive in its set of metrics compared to previous work. We evaluated Type-1 error, FDR control, computation time, AUROC, PRAUC, power, FC bias, and missingness in FDP. We also recommend benchmarking recent/novel DE methods such as glmmTMB and NEBULA, so our benchmarking effort includes these recent DE methods. Our final contribution is to benchmark realistic cell numbers, which contrasts with some previous works [7]. In the future, we expect scRNAseq datasets with high cellular abundances (hundreds of thousands of cells per study) and with multiple samples to be the norm.

In this work, we used two human datasets to study DE method performance. These two datasets were selected based on several criteria. The first criterion used was that we wished to study 10× Genomics data as that is a predominant platform in the field of single-cell analysis. The second criterion was a requirement for the dataset to have high-quality cells obtained from multiple subjects from both healthy and disease conditions, each of which had corresponding metadata that contained covariates that could be used within the simulation study to adjust for subject-level effects. An additional practical consideration was that the data should contain an adequate number of cells per cell type per subject for DE analysis and that the cell types had been annotated within the published data by subject matter experts. Finally, we wished to select datasets relevant to biologically meaningful disease states, so that we could evaluate simulation performance from within a biological context and further evaluate a real data DE analysis to compare our findings to previously published findings. To this end, we selected single-cell data studying multiple sclerosis (MS) [5] and a dataset studying pulmonary fibrosis [6]. Choosing two separate disease areas helps verify the robustness of our conclusions.

MS is a progressive neuroinflammatory disease, which relapses and remits during early stages and develops distinct lesions and neurodegeneration at later stages. MS is related to demyelination and plaque formation [24,25], together with axonal damage in white matter (WM) [26,27], which are usually caused by immune-related cytotoxic damage of oligodendrocytes (OL). The progression of MS’s lesions and its mechanisms in different brain tissues are still not well-understood. Using scRNA-seq to investigate cell-type-specific gene expression can uncover the mechanisms of MS in different tissues and central nervous system (CNS) cell types.

Pulmonary fibrosis is also a progressive disease with the replacement of normal alveolar tissue with connective tissue which reduces the lung’s ability to exchange air [28]. This replacement usually occurs in pathological wound healing, especially during repeated injuries or chronic inflammation [29]. The cause of Pulmonary fibrosis is not well understood. Connective tissue disease, environmental pollution, and infection can cause pulmonary fibrosis [6]. The mechanisms of different cell populations during pulmonary fibrosis progression are poorly understood. Using scRNA-seq data, we can analyze the gene expression in each cell population and uncover their progressive changes.

The rest of the paper is organized as follows. The Materials and Methods section presents our simulator, different DE benchmarking methods, and metrics for evaluating performance. The DEG results on both simulated and real data are in the Results section, where the simulated data has performance comparisons using different metrics, e.g., FDR and AUROC, and the real data are compared using normalized enrichment scores (NES). As a benchmarking paper, we conclude with the performance of different single-cell and pseudo-bulk DE methods and discuss our biological findings in the Conclusion and Discussion sections.

## 2. Materials and Methods

### 2.1. Analysis Datasets

Data from two publications were used for the simulation. The first dataset [5] (PRJNA544731) contained the Schirmer MS snRNA-seq data which profiled grey matter (GM) and white matter (WM) of 12 MS samples at various MS stages and 9 control samples. We re-processed the dataset to include 44,992 cells and 33,525 genes. Cell-type annotation was from the authors, including excitatory neuron (EN) upper layers 2-3 (L2-3), EN-L4, deeper layers EN-L5-6, EN-MIX, inhibitory neuron PVALB (IN-PVALB), IN-VIP, OL, Oligodendrocyte precursor cells (OPC), and astrocytes. After batch correction harmonization across samples done by Harmony [30], and followed by Louvain clustering, we decided to collapse Oligodendrocyte A, B, and C (OL-A/B/C) and Excitatory neuron layer 2/3 A, B (EN-L2-3A/B) to form single aggregated ‘Oligodendrocyte’ (OL) and ‘Excitatory neuron layer 2/3’ (EN-L2-3). The original subclusters were driven by specific donors and no clear subcluster was observed after batch correction. The contrast considered for simulation was between multiple sclerosis (MS) subjects as the disease group and Control subjects as the reference group. The cell types of stomal cells, phagocytes, T cells, and B cells were dropped as there were too few cells after two rounds of filtering. The microglia cell type was also excluded due to an external rule where each cell type must have at least 3 samples per group with at least 10 cells per sample.

The second dataset was obtained from Reyfman 2018 (GSE122960) [6] and was pre-processed to include 77,326 cells and 33,694 genes. Data were collected from 8 lung biopsies (4 idiopathic pulmonary fibrosis, 2 systemic sclerosis, 1 polymyositis, 1 chronic hypersensitivity pneumonitis) and 8 lung explants. The contrast considered was processed to collapse 8 subjects into a single disease group versus 8 donors. Reyfman et al. annotated the cells into cell types of Alveolar type I (AT1), alveolar type II (AT2), alveolar macrophages, and ciliated epithelial cells (referred to as simply ‘ciliated’), F13A1 macrophages, SPP1-macrophages, and SMC + Fibroblasts.

### 2.2. Simulation Methods

Simulations were performed on a per cell type basis for each data set. In total, 9 subjects were chosen from each group in the Schirmer data, and 8 subjects were chosen for each group of the Reyfman data. Fold changes (FCs) and DEG proportions were specified as a priori for different simulation scenarios. For Schirmer data, FCs of 1.2 and 1.5 were simulated setting 20% of genes as DEG for each cell type. For Reyfman data, FCs of 1.4 and 1.5 were simulated setting 15% of genes as DEG for each cell type. Utilizing Reyfman’s simulated data given FC = 1.2, poor performance was observed by all 12 DE methods and made evaluation comparison indistinct (e.g., power close to zero across all DE methods). Null simulations were performed by setting the simulation FC to 1.0 while holding other parameters constant. The simulation process workflow is outlined in Figure S1.

To simulate data, the subject means and dispersions were estimated for each subject within each contrast group for given pilot data while accounting for library size effects. Cell-level read counts were then sampled from a negative binomial distribution given the means and dispersions for each subject for each contrast group. The estimated means and dispersions from real reference subjects from non-zero genes (a gene of a given subject with at least 1 cell with UMI > 0) were directly plugged into a negative binomial distribution to generate synthetic read counts for the reference group.

In contrast, the disease group cell-level count means were estimated from the reference means multiplied by the assumed FC parameter (or divided by the FC parameter) for the simulated DEGs (a random sample of genes based on a preselected proportion of differentially expressed (pDE) genes and FC = 1.0 for the simulated non-differentially expressed genes). A total of 50% of simulated DEGs were upregulated and 50% were downregulated. The disease group cell count dispersions were estimated from synthetic disease group means in a general additive model (GAM) trained by regressing dispersions against means in the real diseased subjects. Genes with all zero counts for a particular subject remained zero in the simulated counts. Covariate effects in the disease group were replaced with reference group covariate values. This simplifying assumption guarantees covariate distributions were the same for reference and disease groups. In special cases where a random row was chosen to be DE but control samples were zero for a particular subject, simulated disease counts were generated from the disease subject’s mean and dispersion rather than the prior procedure described.

For Schirmer data, simulated cells per subject were down-sampled to 50%. For Reyfman data, simulated cells per subject were down-sampled to 25% for Alveolar Macrophage (27.5k cells) and AT2 (26k cells) cell types due to a large number of cells in each of those cell types. Down-sampling was not performed for simulated counts from other Reyfman cell types. The data simulation was well-controlled by constant seed numbers when randomly sampling cell-level counts from the negative binomial distribution, assigning differentially expressed genes, allocating up/down-regulation on the DEGs, choosing arbitrary disease group subjects, and randomly selecting cells per subject for down-sampling.

### 2.3. Diagnostic Plot Methods

**Figure 1:**
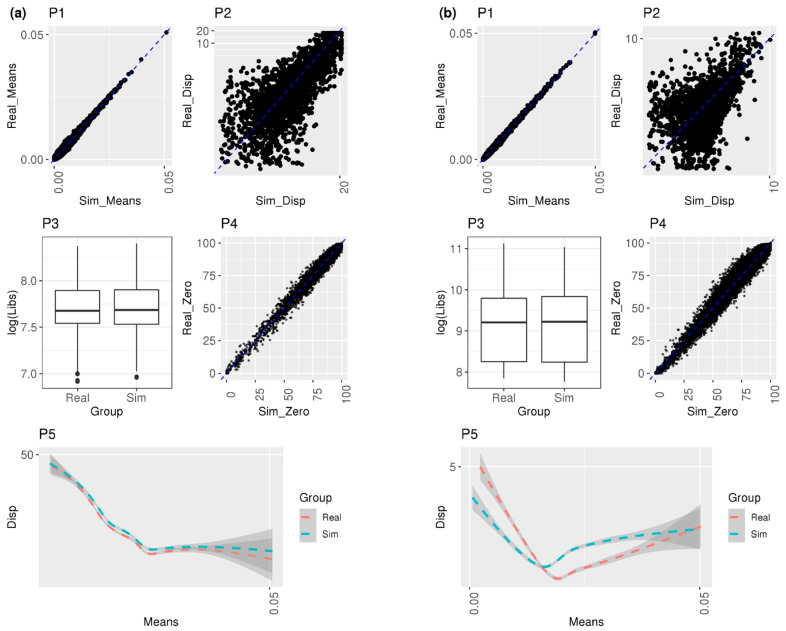
Diagnostic plots to compare the simulation with real data. (**a**) One control sample from EN-MIX cell type in Schirmer et al. [5]; (**b**) one control sample from AT1 cell type in Reyfman et al. [6]. P1: scatterplot of all gene means from real vs. simulated cell-level normalized counts, P2: scatterplot of filtered dispersions from real vs. simulated cell-level normalized counts, P3: boxplot of all library sizes from real vs. simulated cell-level normalized counts, P4: scatterplot of the proportion of zero counts from real vs. simulated cell-level normalized counts, and P5: Loess smoother with 95% confidence intervals of the relationship between the filtered means and dispersions from real vs. simulated cell-level normalized counts.

To validate similarity in distributional aspects from the reference group subjects between real and simulated data sets, means, dispersions, library sizes, and drop-out proportion of cell-level counts from the reference group subjects were visually diagnosed in various plots. Figure 1 illustrates a scatterplot of the all gene means from real vs. simulated cell-level normalized counts, a scatterplot of the filtered dispersions from real vs. simulated cell-level normalized counts, a boxplot of all library sizes from real vs. simulated cell-level normalized counts, a scatterplot of the proportion of zero counts from real vs. simulated cell-level normalized counts, and a Loess smoother with 95% confidence intervals of the relationship between the means and dispersions from real vs. simulated cell-level normalized counts. For each reference group subject, the simulated cell count means and dispersions per gene were driven by the subject-level mean and dispersion. The filtered genes were selected as having dispersion greater than the minimum dispersion for each real and simulated data set. The dispersion for each gene was derived from non-zero read counts where at least one cell contains the non-zero count. An additional analysis was performed to compare our MSMC-Sim simulator performance to default settings for SPsimSeq [9] using the diagnostic plot method (see Figure S9). The intersection of the expressed genes where all cells’ UMI ≥ 2 with dispersion greater than the minimum dispersion value in each real dataset for a cell type and those genes using the same filtering approach that were simulated by both the MSMC-Sim and by SPsimseq was utilized so that the diagnostic results would be comparable in this figure.

### 2.4. DE Benchmarking Methods

DE benchmarking on simulated cell counts was performed using 12 DE methods after applying cell- and gene-level filtering on the simulated data. Filtering occurred in two rounds. The first round of filtering removed mitochondrial genes and cells with library size less than 200 UMI counts or greater than 20 M UMI counts. For the second round of filtering, genes were kept based on a threshold of 10% of the cellular abundance of the smaller of the two groups of a particular cell type. Two types of filtering strategies were used in the second round of filtering. Using ‘or’ logic filtering, the second round of filtering kept genes expressed in at least 10% of cells in either contrast group. Using ‘and’ logic filtering, the second round of filtering requires genes to be expressed in at least 10% of cells in both contrast groups. To account for cell types with small abundances, if 10% of the cell abundance of either group of a particular cell type is below 50 cells, a threshold of 50 cells replaces the 10% threshold. Additional filtering steps include; (1) subjects were dropped from a cell type contrast when fewer than 5 cells remained in the subject after filtering; (2) a cell type contrast was not performed if fewer than 2 subjects remained in either contrast group. All DE methods were benchmarked on the same simulated cell count data.

Eight pseudobulk DE methods were tested: *t*-test, ANCOVA, *u*-test, edgeR [15], limma-voom [16], DESeq2 [17] with internal filtering and without log2FC shrinkage, DESeq2 with internal filtering and with log2FC shrinkage, and DESeq2 without log2FC shrinkage and with internal filtering disabled. Four single-cell methods were tested: glmmTMB (using nbinom2 family function) [18], NEBULA (HL and LN methods) [19], and MAST (with cellular detection rate enabled) [20]. For single-cell DE methods of glmmTMB and NEBULA (LN and HL methods), the library size was included as an offset term and the subject random effect was also modeled. For DE methods of ANCOVA, edgeR, limma-voom, DESeq2 (3 scenarios: without shrinkage and with internal filtering, with shrinkage and with internal filtering, or without shrinkage with no internal filtering), NEBULA (LN and HL methods), glmmTMB, and MAST, *additional covariates were modeled in the design matrix*. For Schirmer data, age, sex, and cell capture batches were included as covariates. For Reyfman data, age and sex were included as covariates.

### 2.5. Simulation Performance Methods

Multiple performance metrics were calculated for each DE method: empirical false-positive rate (empirical FPR), true positive rate (TPR or power), observed false discovery proportion (observed FDP), area under the receiver operating characteristic curve (AUROC), area under the precision-recall curve (PRAUC), absolute FC bias, and computation time. To assess type-I error control, the proportion of DEGs identified at a nominal type-I error rate of 0.05 to the total number of genes analyzed under the null hypothesis was calculated as the empirical FPR. Power was determined under the alternative hypothesis as the proportion of true positive DEGs detected to the total true simulated DEGs using multiple comparison testing at FDR = 0.05. Observed FDP was calculated as the proportion of false positive DEGs detected to the total number of DEGs significantly detected at fixed FDR = 0.05. Two R packages, ROCR [31] and PRROC [32], were employed to calculate AUROC and PRAUC. Absolute FC bias was calculated by taking the absolute difference in DE method identified FC estimates from simulated FC values. The computation time for each simulation data set was measured by the elapsed time in seconds. For each evaluation metric, 12 DE methods were assigned into good/intermediate/poor performance according to specific thresholds. For instance, k-means clustering was applied to classify the DE methods into three groups in power medians: the group containing the highest power median was assigned a good performance; the group involving the lowest power median was assigned a poor performance; otherwise, the performance was assigned as intermediate. For FDR control, good performance was assigned if the observed FDP median fell between 0.0167 and 0.15 with no more than 75% on one side; poor performance was assigned if the observed FDP median was ≥0.25 or ≤0.01 or at least one observed FDP was missing; otherwise, intermediate performance was assigned [7]. For the proportion of missing FDP, good performance was assigned if no missing value of observed FDP exists; intermediate performance was assigned if the proportion of missing observed FDP < 0.5; otherwise, poor performance was assigned (see Table 1 for more details regarding the thresholds for other metrics). The clustering was then color-coded in a heatmap for each cell type: good in blue, intermediate in yellow, and poor in red (see Results Section 3.9).

### 2.6. Real Data DE Application Methods

Real DE analysis was performed on the full set of samples in Schirmer data and in Reyfman data based on the top-performing DE methods according to our simulation study. The filtering settings used were identical to those described in the DE benchmarking methods, using the ‘and’ logic filtering described for the second round of filtering. Covariates and DE formulae used for the real analysis were also identical to those used in the DE benchmarking methods. The cell types selected to analyze in the real data were cell types of relevance from the results of the DE analyses in the Schirmer and Reyfman publications. For the Schirmer data, we chose to perform real DE analysis on the EN-L2-3, EN-L4, OL, and OPC cell types. For the Reyfman data, we chose to perform real DE analysis on the Alveolar macrophage, AT2, and SMC + Fibroblast cell types.

For top-performing DE methods, we first obtained a list of DEGs together with their FDR adjusted *p*-value and log2FC. Upset plots and volcano plots were then generated to compare the three methods and to visualize the up- and down-regulated DEGs. DEGs between disease and control conditions were obtained using DESeq2, glmmTMB, and NEBULA methods separately, because they are the top performing DE methods identified by the simulation exercise (see Results). The cutoff setting is the same as that of simulated data for both datasets, with FDR = 0.05. For DEG overlaps in the upset plots, a further criterion of FC = 1.5 thresholds was applied. After that, we conducted a gene set enrichment analysis (GSEA) using the gene ontology (GO) biological process database (MSigDB version 7.5.1) [33,34], which was also used in the original works as the GSEA database. We adopted Fgsea (https://github.com/ctlab/fgsea, last accessed 22 April 2022) [35], a fast pre-ranked GSEA tool, to perform GSEA analysis. For Schirmer data GSEA analysis, enrichment is considered significant if FDR < 0.05, following the setting in Schirmer’s work [5]. For Reyfman’s data GSEA analysis, enrichment is considered significant if the adjusted *p*-value < 0.01, following the setting in Reyfman’s work [6].

## 3. Results

### 3.1. Diagnostic Plots

Diagnostic plots (P1–P5) in Figure 1a display that distributional characteristics of genes within the simulated reference group subject (C1) are approximately the same as those within the real subject in EN-MIX cells from Schirmer et al.: the scatterplot (P1) shows high concordance in means across all genes; the scatterplot (P2) exhibits that the majority of filtered genes shared the identical dispersions from real vs. simulated data sets; the boxplot (P3) compared log-scale of library sizes and almost identical distributions from both data sets were observed; the scatterplot (P4) shows a strong positive correlation between two data sets in the proportion of zero counts for each gene, and the last plot (P5) exhibits a Loess smoother with 95% confidence interval on the mean/dispersion and a monotonically decreasing relationship between mean and dispersion was observed comparing real vs. simulated data. Interestingly, from P5 in Figure 1a, dispersion from simulated data was consistently higher than real data across means, but the distance is negligible.

The visualization results can vary depending on different subjects or cell types. Using another reference group subject (C4) in the same EN-MIX cell type, the boxplot in Figure S2a shows that the median of simulated library sizes shifted relatively down from the median of real library sizes. Unlike P5 in Figure 1a consistently displaying larger dispersion from simulated data compared to real data, a smaller dispersion from simulated data was observed in comparison to real data when the means were small from P5 in Figure S2a. Nevertheless, the overall investigation of multiple reference group subjects from EN-MIX did not demonstrate a substantial discrepancy between simulated and real data in various distributional features. Likewise, diagnostic plots within Astrocytes cell-type (Figure S2b,c) did not exhibit a severe departure of simulated data from real data.

Figure 1b visualized the distributional similarity across genes in one reference group subject of AT1 cell-type from Reyfman et al. High concordance in the means, library sizes, and drop-out rates were detected from P1, P3, and P4, respectively. On the contrary, compared to Figure 1a, lower concordance was observed in the dispersions (P2) and relationships between mean and dispersion (P5) from Figure 1b. The plots from another reference group subject (Figure S2d) and different SMC + Fibroblasts cell-type (Figure S2e,f) resulted in similar patterns to Figure 1b and Figure 1a, respectively. Figure S9 visualizes the diagnostic plot for the same sample and cell types depicted in Figure 1 for the Schirmer and Reyfman datasets. A difference is observed in the simulated library sizes for the selected combination of sample and cell type in SPsimSeq (plot P3 in Figure S9c,d compared to P3 in Figure S9a,b).

### 3.2. Type-I Error Rate Control

**Figure 2:**
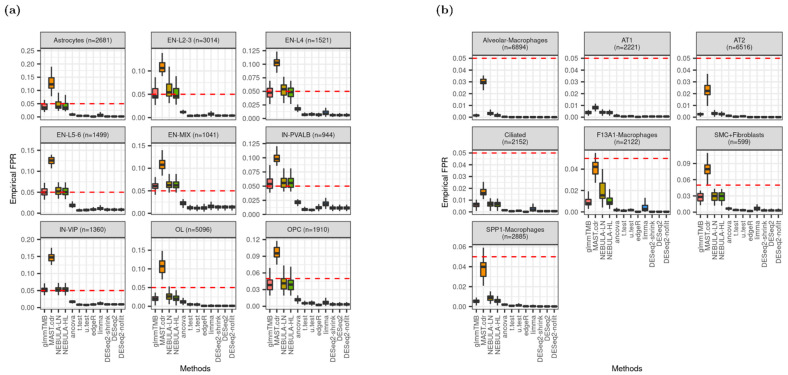
Distribution of empirical false positive rate (FPR) given type-I error rate is 0.05 (red dotted line). (**a**) Data were simulated based on Schirmer et al. [5] and lowly-expressed genes were excluded by ‘and’ filtering scheme; (**b**) data were simulated based on Reyfman et al. [6] and lowly-expressed genes were excluded by ‘and’ filtering scheme.

Under the null hypothesis where no DEG is assumed with FC = 1.0 for every gene, the proportion of falsely rejected genes (empirical FPR) was compared with the nominal type-I error rate fixed as 0.05. Note that a gene was detected as DE if its raw *p*-value < 0.05. From Figure 2a, the empirical FPR medians, which were estimated by glmmTMB and NEBULA, were closest to the nominal type-I error rate from cell types such as EN-L2-3, EN-L4, EN-L5-6, and IN-VIP. For other cell types, inflated or deflated FPR medians were observed, but the medians computed by glmmTMB and NEBULA were consistently much closer to the nominal type-I error rate than other DE methods. Noticeably, MAST.cdr was the most liberal DEG method by showing the highest rejection rate across all cell types in Figure 2. The biggest number of rejected genes enabled MAST.cdr to achieve a median FPR closest to the nominal type-I error rate when empirical FPR from other methods was severely deflated (Figure 2b). When ‘or’ filtering was employed to test more lowly-expressed genes, the smallest deviation by glmmTMB and NEBULA methods from target type-I error rate was again detected for all cell types in Schirmer et al. and SMC + Fibroblasts cell-type in Reyfman et al. (Figure S3).

### 3.3. FC Bias

**Figure 3:**
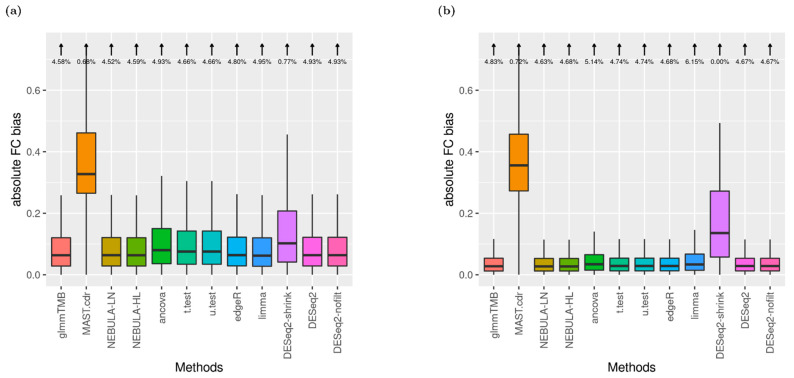
Boxplots of absolute FC bias (arrows denote the proportion of boxplot outliers) given FC = 1.5 and ‘and’ filtering. (**a**) EN-L4 cell type data were simulated based on Schirmer et al. [5]; (**b**) Alveolar-macrophages cell type data were simulated based on Reyfman et al. [6].

To assess FC estimates departing from true (assumed) FC values, absolute FC bias was computed for each DE method where the absolute FC bias equals |FC estimate—true FC|. Figure 3 displays distributions of the metric in addition to the proportion of outliers.

Under the small arrow on top of the boxplot, outliers were defined as the absolute biases greater than the 3rd quartile + 1.5 X IQR (Inter-quantile Range). For EN-L4 cells, most DE methods returned similar distributions with 3~4% outliers proportion excluding MAST.cdr and DESeq2.shrink in Figure S4a. Noticeably, DESeq2.shrink achieved higher consistency in FC estimates as producing no outliers (0% from Figure S4a). Most observations are similar from Figure S4b compared to Figure S4a. However, bigger variances for MAST.cdr and DESeq2-shrink were detected, and the outlier’s proportions became higher (4~6%) across the other ten DE methods from Alveolar-macrophages cells in Reyfman et al. When FC was increased to 1.5, the same rankings among DE methods in the absolute bias of FC estimates were displayed (Figure 3).

### 3.4. FC Correlations

**Figure 4:**
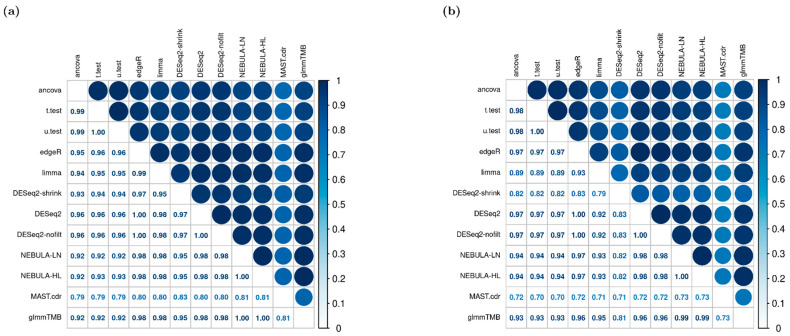
Pairwise correlation matrix of FC estimates given FC = 1.5 and “or” filtering scheme. (**a**) EN-MIX cell type was simulated based on Schirmer et al. [5]; (**b**) SMC + Fibroblasts cell type was simulated from Reyfman et al. [6].

Pairwise comparison of FC estimates among the twelve DE methods was measured by Spearman’s rank correlation coefficient (rho). Figure 4 includes the correlation coefficient matrices calculated from Schirmer et al. and Reyfman et al datasets. Across the two data sets, it was commonly observed that FC estimates derived by either NEBULA-LN or NEBULA-HL were almost the same as those by glmmTMB (rho≈1). Moreover, it was noticeable that FC estimates by edgeR, DESeq2, and DESeq2 with no internal filtering were closer to NEBULA or glmmTMB than other pseudo-bulk DE methods such as ANCOVA, *t*-test, and *u*-test (rho < 0.95). As Figure 3 shows the greatest absolute bias in FC estimates by MAST.cdr and DESeq2.shrink, FC estimates by those two DE methods evaluated the lowest correlation coefficients with other DE methods in Figure 4b (rho < 0.85). The pairwise comparison did not include genes with at least one missing FC among the twelve FC estimates.

### 3.5. FDR Control

**Figure 5:**
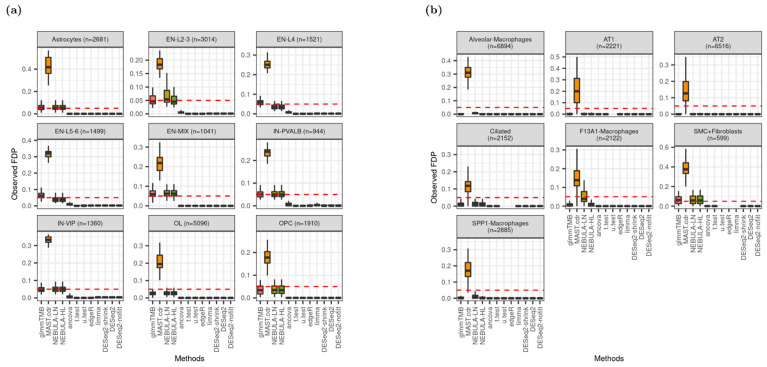
Distribution of observed false discovery proportion (FDP) given FC = 1.5 and ‘and’ filtering scheme at nominal FDR = 0.05 (red dotted line). (**a**) Data were simulated based on Schirmer et al. [5]; (**b**) Data were simulated based on Reyfman et al. [6].

Figure 5 displays the distribution of observed false discovery proportion (FDP) and suggests that glmmTMB and NEBULA control FDR better than other DEG methods because the median FDP is closest to the nominal FDR, 0.05. These methods showed the closest proximity to the nominal FDR in the Schirmer data simulations in Figure 5a. Although pseudobulk DE methods failed to control FDR by showing severely deflated FDP across all cell types, MAST.cdr resulted in high inflation of FDP above the fixed FDR. Similarly, Figure 2a depicted failures of Type-I error rate control due to MAST.cdr’s inflation and pseudo-bulk DE methods’ deflation. The figures can be explained in which MAST.cdr tends to select many false positives while pseudobulk DE methods are too conservative to detect true DEG. From Figure 5b, some pseudo-bulk DE methods returned all missing observed FDP over 50 simulation data sets (e.g., u.test for AT1 cell-type). The missingness (Figure 5b) mostly happened when a DEG method could identify no DEG (i.e., zero power in Figure S6a). As more lowly-expressed genes were tested, pseudo-bulk DE methods produced more missing observed FDP except for Alveolar-Macrophages, AT2, and SMC + Fibroblasts cell types (Figure S5).

### 3.6. Power

**Figure 6:**
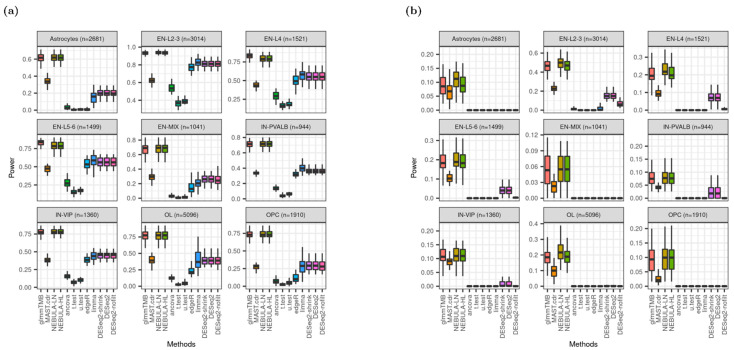
Boxplots of power given “and” filtering scheme based on Schirmer et al. [5]. (**a**) when FC = 1.5; (**b**) when FC = 1.2.

Power (true positive rate, TPR) is defined by a proportion of true DEG detected over true DEG as simulated. In this experiment, the Benjamini-Hochberg method which is a classical multiple-comparison testing procedure, adjusted the raw *p*-value of each gene. The adjusted *p*-value was compared with 0.05 of the nominal FDR to call DEGs. In Figure 6, glmmTMB and NEBULA cell-level methods outperformed MAST.cdr and pseudo-bulk DE methods as achieving higher power regardless of FC magnitudes assumed. Noticeably, when independent filtering embedded in DESeq2 was inactivated (DESeq2-nofilt), the power from regular DESeq2 (DESeq2) was substantially diminished for most cell types in Figure 6b. The decrease in power implies that DESeq2 and the shrinkage version of DESeq2 (DESeq2-shrink) tested fewer lowly-expressed genes and calculated power after the additional independent filtering, which was not employed in other DE methods.

### 3.7. AUROC and PRAUC

**Figure 7:**
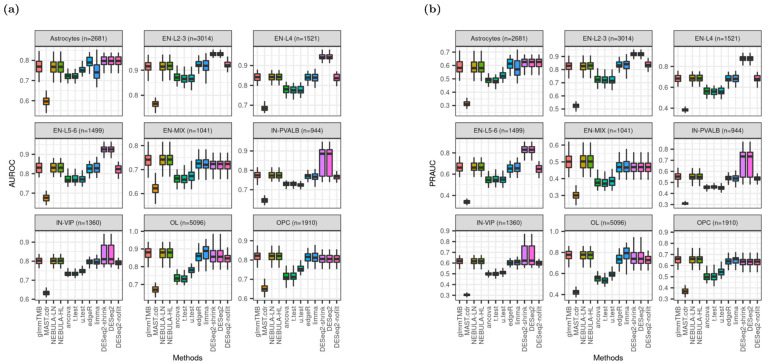
Boxplots of AUROC and PRAUC given FC = 1.2 and ‘or’ filtering scheme from Schirmer et al. [5]. (**a**) AUROC; (**b**) PRAUC.

Figure 7 shows better rankings of DESeq2.shrink and DESeq2 in medians of AUROC and PRAUC for the cell types such as Astrocytes, EN-L2-3, EN-L4, EN-L5-6, and IN-PVALB. However, the metrics from DESeq2_nofilt were dramatically decreased and are comparable to those from glmmTMB or NEBULA cell-level methods. Moreover, for cell types of IN-PVALB and IN-VIP, the distribution of AUROC or PRAUC from DESeq2 is more widely spread out than glmmTMB and NEBULA. All medians of AUROC (PRAUC) from glmmTMB and NEBULA cell-level methods were greater than 0.75 (0.5) across different cell types, demonstrating high accuracy to identify true DEGs. In terms of AUROC and PRAUC performance from the Reyfman et al. data set (Figure S7), DESeq2 and edgeR were in the first place with higher values. However, the difference is very small from other DE methods in the second decimal place, such as the glmmTMB and NEBULA methods.

### 3.8. Computation Time

**Figure 8:**
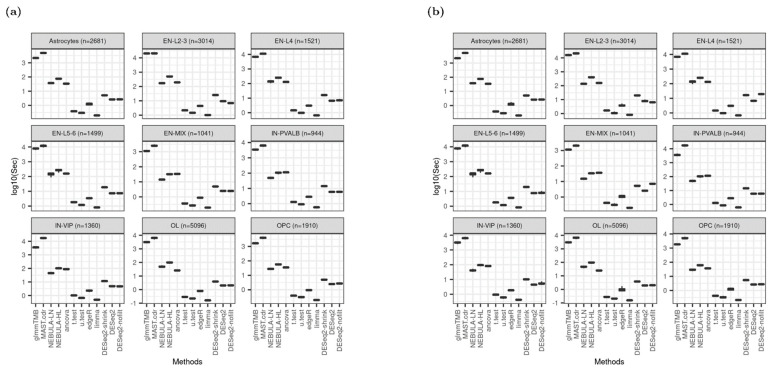
Boxplot of elapsed time in logarithmic scale at base 10 based on Schirmer et al. [5] when FC = 1.5. (**a**) Using ‘and’ filtering scheme; (**b**) Using “or” filtering scheme.

MAST.cdr and glmmTMB implementation consume much more computational burden: >1000 s to run each simulation data set on average. Although NEBULA methods are cell-level methods like MAST.cdr or glmmTMB, they took substantially less time with <300 (≈10^2.5^) seconds for each simulation, implying that NEBULA methods were three times faster than glmmTMB or MAST.cdr overall. Computation by NEBULA-HL was consistently longer than NEBULA-LN. Pseudo-bulk DE methods except for ANCOVA, are relatively time-efficient compared to cell-level methods in Figure 8. The rankings are identical across all cell types and are not affected by a larger number of lowly expressed genes in the DEG testing set (Figure 8b).

### 3.9. Heatmap

**Figure 9:**
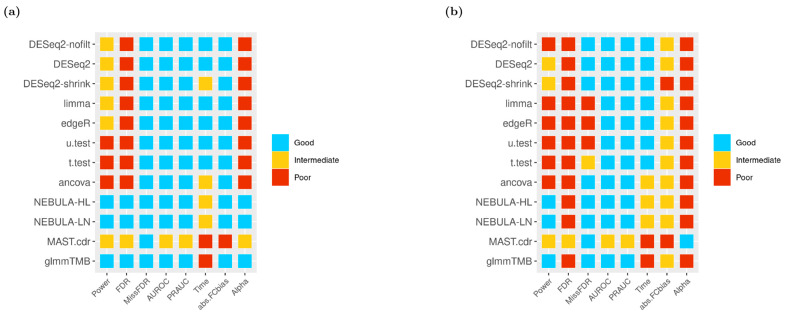
Heatmaps of 12 DE methods in a variety of overall performance metrics given FC = 1.5 using ‘and’ filtering scheme (**a**) EN-L2-3 cell type from Schirmer et al. [5]; (**b**) SPP1-Macrophages cell type from Reyfman et al. [6].

Based on the clustering thresholds described in the Methods section (Table 1), color-coded heatmaps across all DE methods and performance evaluation metrics for specifically EN-L2-3 and SPP1-Macrophages cells are presented in Figure 9. By executing stringent ‘and’ filtering in Figure 9a, both NEBULA-HL and NEBULA-LN methods showed superior performance in most metrics except for time efficiency. In contrast to the other cell-level methods like MAST.cdr or glmmTMB, NEBULA’s running time was comparatively efficient (see “Time” column in Figure 9). In Figure 9b, most DE methods struggled more to identify true DEG overall and produced more intermediate/poor performance than Figure 9a. For instance, NEBULA-HL failed to control FDR and Type-I error rate in Figure 9b, whereas the method is successful in Figure 9a. Nevertheless, NEBULA methods and glmmTMB achieved relatively high power and derived no missing FDP. Moreover, higher AUROC and PRAUC than MAST.cdr were consistently observed by NEBULA methods and glmmTMB (Figure 9b). Additional heatmaps from other cell types such as Astrocytes, EN-MIX, and IN-VIP from Schirmer et al. and Ciliated, SMC + Fibroblasts, and Alveolar macrophages from Reyfman et al., are displayed in Figure S8a–f. Consequently, the overall performance evaluation in heatmaps illustrated the superior performance of glmmTMB and NEBULA in power, FDR control, and type-I error control and superior performance of DESeq2 in AUROC and PRAUC.

### 3.10. Real Data Application Results

**Figure 10:**
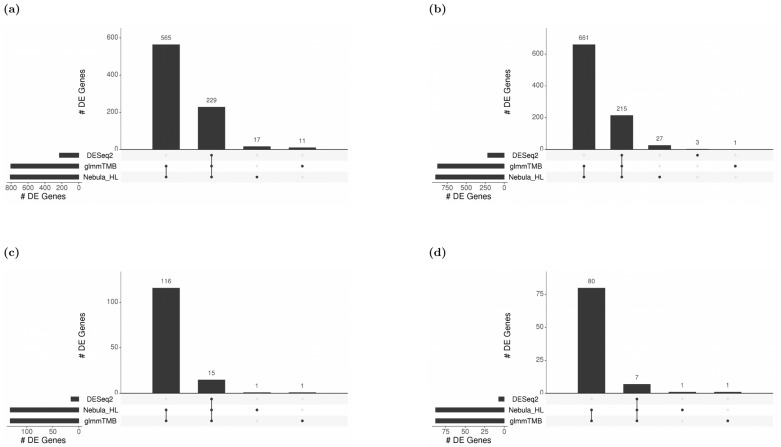
Upset plots showing the overlap of real-data DEGs identified by three DE methods (DESeq2, Nebula HL, and glmmTMB) at FC 1.5 and FDR 0.05 cutoffs for Schirmer data. Upset plot overlap is shown per cell type. (**a**) Overlap of DEGs for EN-L2-3 cells, (**b**) Overlap of DEGs for EN-L4 cells, (**c**) Overlap of DEGs for OL cells, and (**d**) Overlap of DEGs for OPC cells.

**Figure 11:**
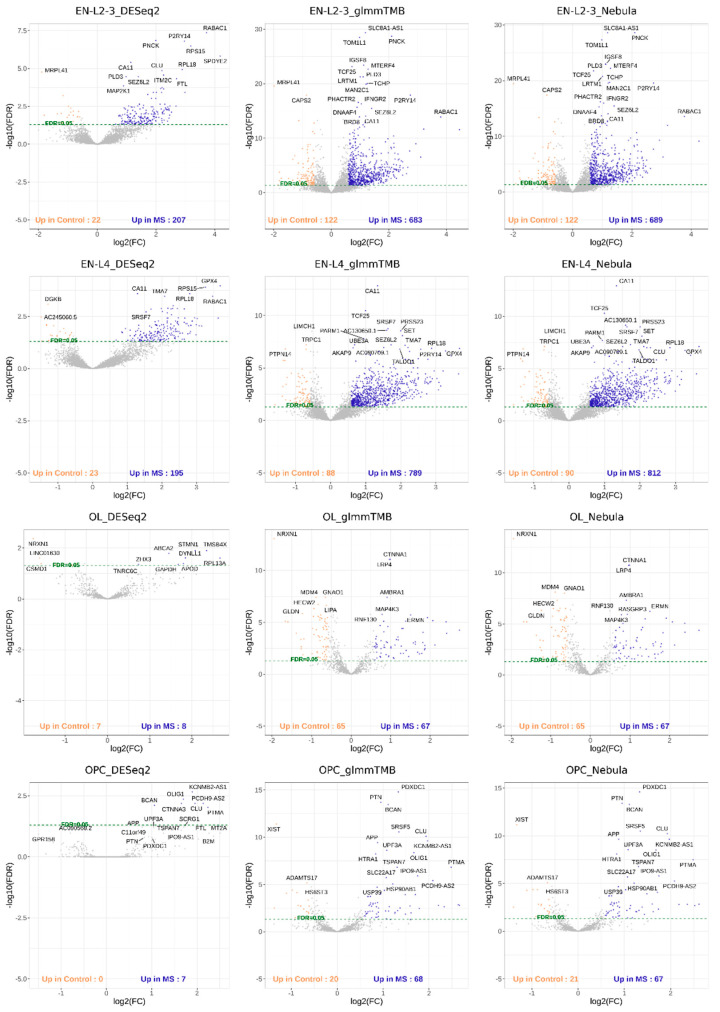
Volcano plots of EN-L2-3, EN-L4, OL, and OPC cell types for Schirmer data [5].

**Figure 12:**
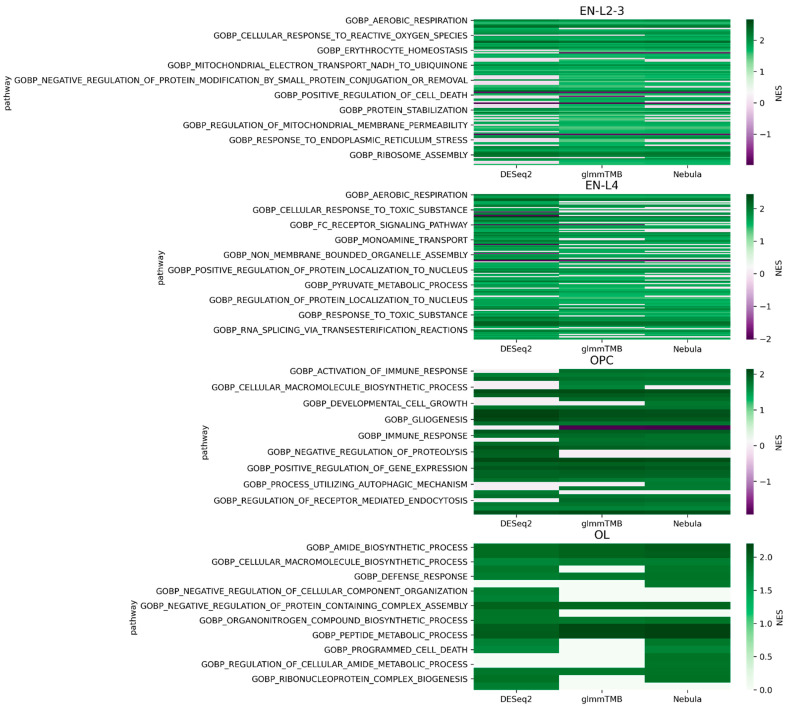
Heatmap plots of EN-L2-3, EN-L4, OL, and OPC cell types for Schirmer data [5].

For each cell type of interest in the Schirmer data, upset plots (Figure 10) and volcano plots (Figure 11) were generated to compare the three DE methods and the up- and down-regulated DEGs. From Figure 10, the results of NEBULA-HL and glmmTMB are similar, with highly overlapped DEG lists and comparable list size, while DESeq2 detected much fewer DEGs. Volcano plots (Figure 11) also demonstrate that NEBULA-HL and glmmTMB were comparable in terms of DEG list size while DESeq2 detected much fewer DEGs. Moreover, both NEBULA-HL and glmmTMB detected dysregulation of PPIA (Peptidylprolyl isomerase A) and CUX2 (a marker of supragranular layers) [36], two important genes validated in Schirmer’s work using a smFISH experiment. PPIA is a disease modifier that is a translational biomarker for amyotrophic lateral sclerosis and is associated with frontotemporal lobar degeneration [37]. EN CUX2 upper cortical neurons are more vulnerable to meningeal-driven oxidative stress and cell death [5]. The failure of DESeq2 in detecting PPIA and CUX2 further demonstrated the superiority of NEBULA-HL and glmmTMB. A heatmap plot (Figure 12) of the enriched GO terms was generated to compare the three DEG methods. Higher NES indicates that the GO term is more over-represented. More rows of NES = 0 indicates the worse performance of the DEG method. From the heatmap (Figure 12), the DEGs detected by NEBULA-HL enriched the most GO terms.

We selected the highly MS-related GO terms (Table S1–S3) and compared the results to the findings in Schirmer’s work [5]. Only EN-L2-3, EN-L4, and EN-L5-6 were included for comparison as Schirmer’s work only reported the GO enrichment results of these three cell types. Schirmer et al. conducted two versions of DEG analyses: 1. a pseudo-time trajectory DEG analysis in upper cortical layer EN-L2-3 using control, acute, and chronic MS patients; 2. a regular DEG analysis of MS versus controls. EN-L2-3 was used for trajectory analysis because its demyelination and the number of cells showed a high correlation with the pseudo-time progression. We detected the dysregulation of protein folding and protein targeting in upper neuron layers EN-L2-3, which is consistent with the findings in Schirmer’s regular DEG enrichments. On the other hand, our work detected the dysregulation of cell death and oxidative phosphorylation, which were found in Schirmer’s pseudo-time trajectory DEG enrichment. The dysregulation of oxidative stress [38,39] and cell death in upper cortical layers might be a cause of MS. In addition, we found dysregulations of response to a toxic substance in EN-L2-3 and L4, and response to the virus in L4, which suggested MS patients might have had infections [40] in neurons. Pseudo-time trajectory DEG analysis provides an additional view of the transcriptomic changes during different disease stages but has more prerequisites such as progressive disease and additional labeling of disease subtypes or stages. By only using regular DEG analysis, our work detected pathways both in Schirmer’s regular DEG results and their pseudo-time trajectory DEG results, as well as new findings of dysregulation in response to toxic substances and viruses. This demonstrates the outperformance of our DEG methods over conventional methods.

**Figure 13:**
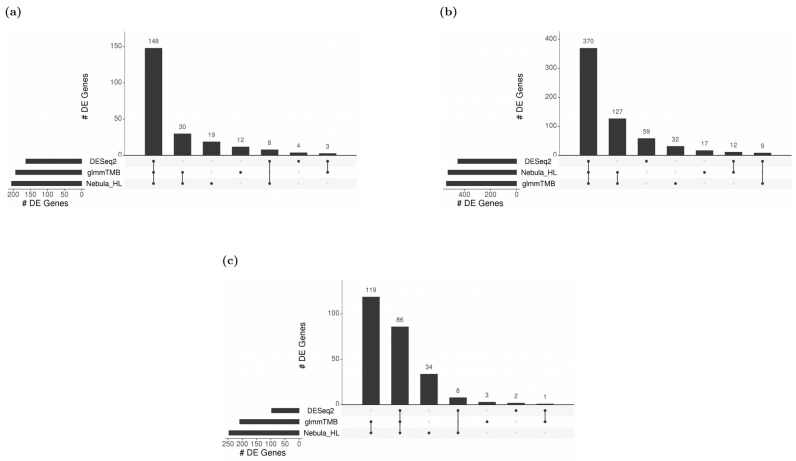
Upset plots showing the overlap of real-data DEGs identified by three DE methods (DESeq2, Nebula HL, and glmmTMB) at FC 1.5 and FDR 0.05 cutoffs for Reyfman data [6]. Upset plot overlap is shown per cell type. (**a**) Overlap of DEGs for Alveolar macrophage cells; (**b**) Overlap of DEGs for AT2 cells; (**c**) Overlap of DEGs for SMC+ Fibroblast cells.

**Figure 14:**
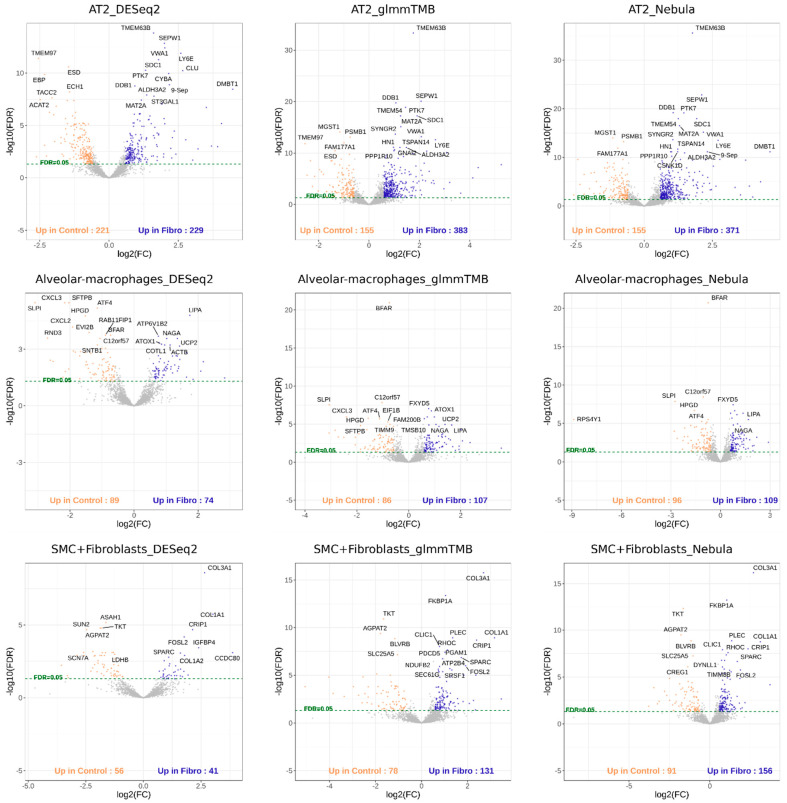
Volcano plots of AT2, Alveolar macrophages, SMC + Fibroblasts cell types for Reyfman data [6].

**Figure 15:**
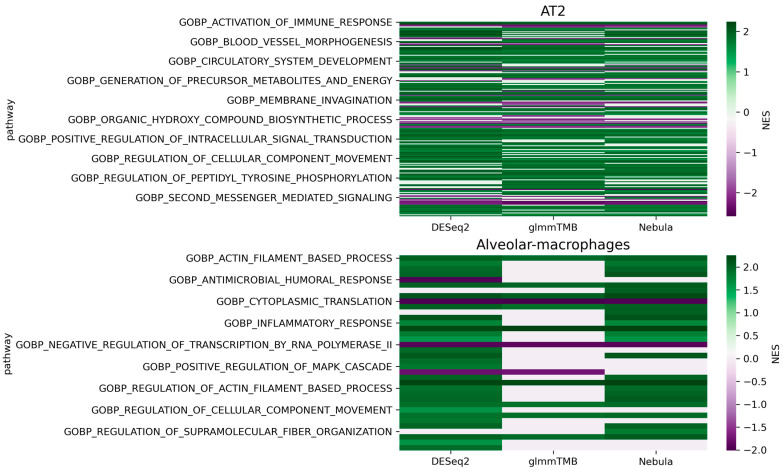
Heatmap of the enriched GO terms (Reyfman Fibrosis data [6]). Color bar indicates normalized enrichment score (NES). Higher NES absolute value indicates more significant; NES = 0 indicates that pathway cannot be enriched. The more rows with NES = 0, the worse performance of the DEG method.

For real Reyfman data analysis for each cell type of interest, we generated upset plots (Figure 13) and volcano plots (Figure 14) to compare the three DEG methods for up- and down-regulated genes. From Figure 13, DESeq2 can find a comparably sized DEG list, and the overlaps between the three DEG methods are high. NEBULA-HL and glmmTMB are still relatively more similar, with larger overlaps. A heatmap plot (Figure 15) of the enriched GO terms was generated to compare the three DEG methods. From the heatmap (Figure 15), the DEGs detected by NEBULA-HL enriched the most GO terms. We selected the highly Fibrosis-related GO terms and compared the result of the two important cell types (alveolar-macrophages, AT2) [6] (Tables S4 and S5) to that in Reyfman’s work.

Compared to Reyfman’s findings, our results (Tables S4 and S5) detected dysregulation of immune-related GO terms in AT2, including activation of immune response, cell activation involved in immune response, immune effector process, innate immune response, leukocyte medicated immunity, as well as inflammatory response, wound healing, and response to wounding. Research [41] has shown that immune, inflammation, and wound healing were related to pulmonary fibrosis. The injury and wound healing in AT2 are early causes of pulmonary fibrosis [42], while immune and inflammation might be secondary features [43]. The dysregulation of these wound healing, immune and inflammation-related transcriptions suggests that the fibrosis lungs might have undergone injuries and healing.

## 4. Discussion

In this manuscript, we presented a novel approach for the simulation and benchmarking of multi-subject multi-condition single-cell/single nuclear RNA-seq datasets. Our simulator differs from previous approaches by focusing on the multi-subject scenario, accounting for covariate effects, flexibly modeling the mean/dispersion relation with GAM, and capturing subject-to-subject variation. The diagnostic plots illustrate that our simulator recapitulated distributional characteristics of real data including average expression, expression dispersion, mean/dispersion relationship, and dropout proportion.

Applying our simulator to the MS dataset from Schirmer et al. and the lung fibrosis data from Reyfman et al., we benchmarked 12 DE methods spanning pseudo-bulk and single-cell DE methods and assessed their performance by utilizing a comprehensive set of metrics (type-1 error rate control, computation time, AUROC, PRAUC, FDR control, power, absolute FC bias, FC correlation, and others). Results from this benchmarking study demonstrated the superior performance of NEBULA-HL for the multi-subject multi-condition scenario (shown in heatmaps: Figure 9 and Figure S8). In detail, NEBULA-HL showed a good overall performance for the metrics of statistical power, AUROC, and PRAUC. In terms of type-1 error rate and false discovery rate control, NEBULA-HL controlled these metrics near the nominal rate for the MS data but showed deflation for the lung data. The deflation was observed when the cell-to-cell variation was crucially small for both control and disease subjects within specific cell types such as Alveolar-macrophages, AT1, AT2, and Ciliated. Computation time for NEBULA-HL was reasonable. GlmmTMB and MAST were typically 1–2 orders of magnitude slower than NEBULA, while pseudo-bulk methods were faster than NEBULA.

Extensive investigation of heatmaps across all cell types recommended NEBULA methods that derived superior performance in the most evaluation metrics: the highest detection power from both data sets (Figure 6 and Figure S6), the closest observed FDP and empirical FPR to the target FDR and target type-I error rate from Schirmer et al. (Figure 2a, Figure 5a, Figures S3a and S5a), respectively. Some pseudo-bulk DE methods such as DESeq2 and its variants achieved the highest AUROC and PRAUC for most cell types (Figure 7 and Figure S7). However, medians of AUROC and PRAUC from NEBULA methods were very close to those from DESeq2 for most cell types. More importantly, severe deflations of observed FDP and empirical FPR, hence failures of FDR and type-I error rate controls across all cell types, made an unfavorable decision of the pseudo-bulk DE methods (Figure 2, Figure 5, Figures S3 and S5).

Between two NEBULA methods (LN and HL), many evaluation metrics exhibited high concordance from most cell types (e.g., similar medians of observed FPR in Figure 2a, identical distribution of absolute FC bias in Figure 3, correlation coefficient close to 1 in Figure 4). Interestingly, performance by glmmTMB showed more similar results to NEBULA-HL than NEBULA-LN (e.g., observed FPR in Astrocytes/EN-L2-3/EN-L4/OL/OPC cell types from Figure 2a and cell types in Reyfman et al. excluding SMC + Fibroblasts from Figure 2b). The similarity is plausible because glmmTMB and NEBULA-HL were developed on a basis of the same underlying distribution, a negative binomial mixed model while NEBULA-LN leverages Large Number approximation to enhance computational efficiency. For robustness, we would recommend NEBULA-HL unless the computation time is unrealistic.

Depending on the goal of the DE analysis, the choice of genes to analyze in a single-cell experiment will rely on careful evaluation of the experimental data. Gene-level filtering for discovery may involve using a looser criterion to retain genes that are lowly expressed, whereas gene-level filtering for the purposes of this study requires stricter filtering to evaluate genes consistently expressed between contrast groups. Similarly, there should be careful consideration given to the handling of rare cell types, which may contain inadequate numbers of cells per sample to perform DE analysis.

Other important considerations when choosing a DE method for multi-sample multi-condition datasets are package usability and complex experimental design support (Table 2). For most DE methods, ample documentation is provided containing example files, reference documentation, quick start guides, and vignettes. In terms of covariate support, all methods except for *t*-test/*u*-test support covariate adjustment. More complex designs involving fixed effect design matrices are supported by most DE method packages while general random effect design matrices are supported by only MAST and glmmTMB.

Our methodology for simulation and benchmarking has a few key limitations. First, our simulation approach may not capture the correlation structure of real data. This includes across-cell type correlations, gene-gene correlations, and correlations of cells within the same cell type of the same subject. Furthermore, none of our simulation diagnostic metrics assessed the recapitulation of real-life correlations. Another important limitation occurs in the modelling of covariates in our simulator; we used a simplifying assumption that covariate distributions in control and disease groups would be similar. Thirdly, our conclusions for our simulator and benchmarking have only been assessed on the 10× Genomics platform. Further research is needed to confirm our conclusions hold for other platforms.

We also need to be mindful of genes that are reasonably expressed in one group but have near-zero expression in the other group. Determining DEGs for this subset of genes is challenging since DE algorithm modelling assumptions may no longer hold true. An alternative approach such as comparing the proportion of expression across groups could be employed. Although it is beyond the scope of this discussion, there are methods that attempt to perform this type of proportional zero binary expression analysis [44]. Special attention should be paid to the identities of genes that fall into this category when considering DEG results as their results may be unreliable.

Some of our key findings differ from previous work in the literature. For example, our benchmarking findings illustrate that cell-level methods such as NEBULA and glmmTMB outperformed pseudo-bulk DE methods overall. These findings contradict the work of [14,45], who claim pseudo-bulk methods outperform cell-level DE methods.

Comparing Squair et al. [14] to our present work, their work assumed bulk RNA-seq differential expression as the ground truth and quantified DE concordance between bulk and single-cell expression with the AUCC metric, whereas, in the present study, we assumed simulated DEGs in our multi-subject multi-condition simulator as the ground truth. Additionally, Squair et al. did not consider recent single-cell approaches such as glmmTMB or NEBULA in their benchmarking study. As for benchmarking metrics, this present work considers many more evaluation metrics (type-1 error control, computation time, AUROC, PRAUC, fdr control, power, usability, and FC bias) compared to Squair’s benchmarking metrics (computation time, AUCC, number of false positives, etc.).

Murphy et al. [45] performed a reanalysis of the work from Zimmerman et al. [46] by considering the Matthews correlation coefficient (MCC) metric, allowing the user to choose the proportion of DEGs, and ensuring the same simulated datasets were utilized across all DE methods. Zimmerman et al.’s results advocate for mixed model methods over pseudo-bulk methods, but the reanalysis of Murphy et al. claims pseudo-bulk methods have the best performance.

Our present work differs from Murphy et al. in several ways. First, our simulator uses raw counts as input and simulates raw counts as output. Both Murphy et al. and Zimmerman et al. expect TPM input data and simulate TPM level data. Consequently, our work uses simulated raw counts as input to the 12 DEG methods but Murphy and Zimmerman utilize TPM as DE method input. Other important differences include no covariate adjustment during the benchmarking of Murphy et al. and Zimmerman et al. and different simulator assumptions.

Application of the DE methods to real data for both the Schirmer and Reyfman datasets demonstrates that two scRNA-seq DEG methods, NEBULA and glmmTMB, outperformed DESeq2 in terms of DEG detected and the subsequent gene set enrichment. NEBULA was slightly better than glmmTMB, according to the DEG results and pathway NES heatmaps. Our DEG analysis detected GO terms both in Schirmer’s conventional DEG result and their pseudo-time trajectory transcriptomic result, which further showed the superiority of our DE pipeline.

The result of this study shows that, despite the limitations inherent in the simulation of scRNA-seq count data, the current simulation results support the use of mixed models for DE (in contrast to previous simulation studies [14,45]). Pseudobulk DE methods tend to be too conservative in the results we studied, as observed in their deflated FDP and empirical FPR. Consequently, pseudobulk DE methods may lose power to detect DEGs, and we observe some evidence of that in our real data application. Lastly, the computational burden of mixed-model methods like glmmTMB is a reasonable obstacle, as has been previously acknowledged [14,45]; however, NEBULA offers a significantly improved runtime and lower computational burden for a mixed-model single-cell DE method. Future work for this study has several possibilities. For the simulation effort, we would like to include additional parameters to simulate FC distributions and an imbalanced contrast group design as opposed to requiring simulation of equal numbers of disease and control samples as in the present study. We would also like to enable the simulation of real covariates from input data for disease samples rather than using the current control covariate subject-mapping approach. Correlation structures, both between cell types and between individual cells, could also be modelled in future simulations. We would also like to incorporate into our simulator and DE pipeline codes the ability to ingest other single-cell sequence data formats to broaden the datasets capable of being studied using this simulation workflow.

## 5. Conclusions

A broad internal investigation of all simulation scenarios (various cell types, different FC values, two types of filtering strategies, and two data sets) including both Figure 9 and Figure S8 suggests that cell-level DE methods originating from negative binomial mixed models such as glmmTMB and NEBULA-HL outperformed MAST.cdr and pseudo-bulk DE methods on average. Moreover, the primary merit of running NEBULA-HL over glmmTMB is time efficiency.

## Figures and Tables

**Table 1:** DEG assessment metrics clustered into good, intermediate, and poor performance.

	Good	Intermediate	Poor
Power.median	Kmean class including max. median power	Otherwise	Kmean class including min. median power
FDP.median	no more than 75% of FDPs (False Discovery Proportion) on one side (above or below) of 0.05 and 0.0167 < median FDP < 0.15	Otherwise	median FDP ≥ 0.25 or median FDP ≤ 0.01 or at least one FDP is missing
missFDP	0	<0.5	≥0.5
AUROC.median	≥0.9	0.7≤ and <0.9	<0.7
PRAUC.median	≥0.8	0.4≤ and <0.8	<0.4
FPR.median	$\left|log_{2}\left(\frac{median\;FPR}{0.05}\right)\right|<log_{2}\left(1.5\right)$	$log_{2}\left(1.5\right)\leq \left|log_{2}\left(\frac{median\;FPR}{0.05}\right)\right|<2$	$2\leq \left|log_{2}\left(\frac{median\;FPR}{0.05}\right)\right|$
Time.median	≤10	10< and ≤500	>500
Abs(FC bias.median) ($FC^{w}$ is 1.2 for Schirmer et al. [5] or 1.4 for Reyfman et al. [6])	$\leq 0.05\times \frac{FC}{FC^{w}}$	$0.05\times \frac{FC}{FC^{w}}<and\;\leq 0.10\times \frac{FC}{FC^{w}}$	$>0.10\times \frac{FC}{FC^{w}}$

**Table 2:** Comparison of DEG approaches for covariate handling, documentation, and complex design support.

Method	Covariates?	Documentation?	Fixed Effect Matrix?	Random Design Matrix?	Download Link
*t*-test	No	Textbook	No	no	N/A
*u*-test	No	Textbook	No	no	N/A
ancova	Yes	Textbook	No ^1^	no	N/A
edgeR	Yes	vignette, users guide, reference	yes	no	https://bioconductor.org/packages/release/bioc/html/edgeR.html (last accessed 22 April 2022)
limma	Yes	quickstart, users guide, reference	yes	no	https://bioconductor.org/packages/release/bioc/html/limma.html (last accessed 8 February 2022)
DESeq2	Yes	quick start, users guide, reference	yes	no	https://bioconductor.org/packages/release/bioc/html/DESeq2.html (last accessed 11 February 2022)
MAST	Yes	intro, MAST examples, reference	yes	yes	https://www.bioconductor.org/packages/release/bioc/html/MAST.html (last accessed 10 February 2022)
glmmTMB	Yes	multiple vignettes and reference	yes	yes	https://cran.r-project.org/web/packages/glmmTMB/index.html (last accessed 1 April 2022)
NEBULA	Yes	vignette and reference	yes	no	https://cran.r-project.org/web/packages/nebula/index.html (last accessed 2 June 2022)

^1^ Traditional ancova is a single group effect with one or more covariates. However, more complex designs are possible with ancova.

## Data Availability

Not applicable.

## Supplementary Materials

The following supporting information can be downloaded at: https://www.mdpi.com/article/10.3390/life12060850/s1, Figure S1: A flowchart representation of DE simulation and DE method performance evaluation methods; Figure S2: Diagnostic plots; Figure S3: Distributions of observed false positive rate (FPR) given type-I error rate is 0.05 (red dotted line); Figure S4: Boxplots of absolute FC bias given “or” filtering scheme with lower FC; Figure S5: Distribution of observed false discovery proportion (FDP) given a fixed FDR is 0.05 (red dotted line) given “or” filtering scheme and lower FC; Figure S6: Distribution of power over 50 simulation data sets based on Reyfman et al.; Figure S7: Distribution of AUROC and PRAUC over 50 simulation data sets based on Reyfman et al. given FC = 1.4 and lowly-expressed genes were excluded by “or” filtering scheme; Figure S8: Heatmaps of 12 DE methods in a variety of overall performance metrics; Figure S9: Diagnostic plots to compare simulation with real data using different simulation methods; Figure S10: Boxplot of elapsed time in logarithmic scale at base 10 based on Reyfman et al. when FC = 1.5; Table S1: Enriched GO terms for cell type EN-L2-3 (Schirmer data); Table S2: Enriched GO terms for cell type EN-L4 (Schirmer data); Table S3: Enriched GO terms for cell type EN-L5-6 (Schirmer data); Table S4: Enriched GO terms for cell type Alveolar-macrophages (Reyfman data); Table S5: Enriched GO terms for cell type AT2 (Reyfman data).

## Code Availability Statement

The source code written in R 3.5.1 to run simulations and DE analyses is available at https://github.com/interactivereport/scRNAseq_DE (last accessed 27 May 2022). The simulation code was written as a function that allows the user to produce the simulation performance metrics discussed for the datasets analyzed in this study and for user-input data. Similarly, the DE pipeline was written to perform gene- and cell-level filtering and DE analysis for datasets analyzed in this study and user-input data.
