# Supplementary material for: Recommendations of scRNA-seq Differential Gene Expression Analysis Based on Comprehensive Benchmarking
Source: Life (Basel). 2022 Jun 7;12(6):850. doi: 10.3390/life12060850 (PMC9225332; doi:10.3390/life12060850)
Supplement: Supplementary file 1 [file life-12-00850-s001.zip › life-1717524-supplementary.pdf]

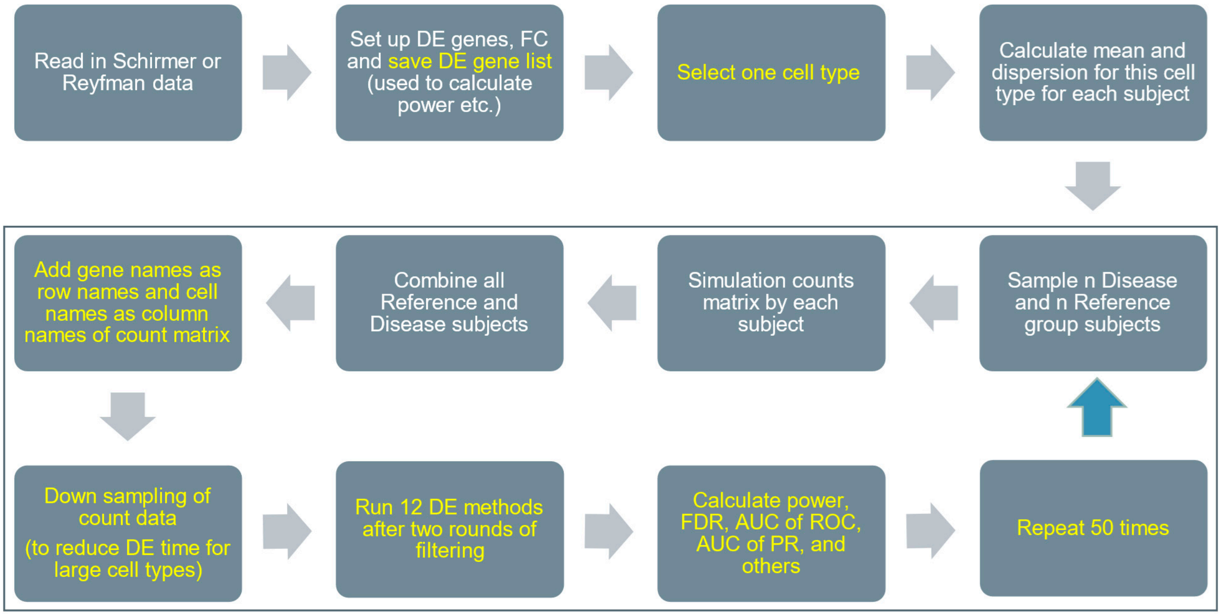

Figure S1. A flowchart representation of DE simulation and DE method performance evaluation methods. Each filled gray text box indicates a separate step in the process of generating simulated counts, performing DE, and evaluating DE performance. The steps in the outlined box indicate the steps that are iterated over during the individual simulations. [Note that the "simulation count matrix by each subject" step and the "run 12 DE methods" step accounts for covariates]

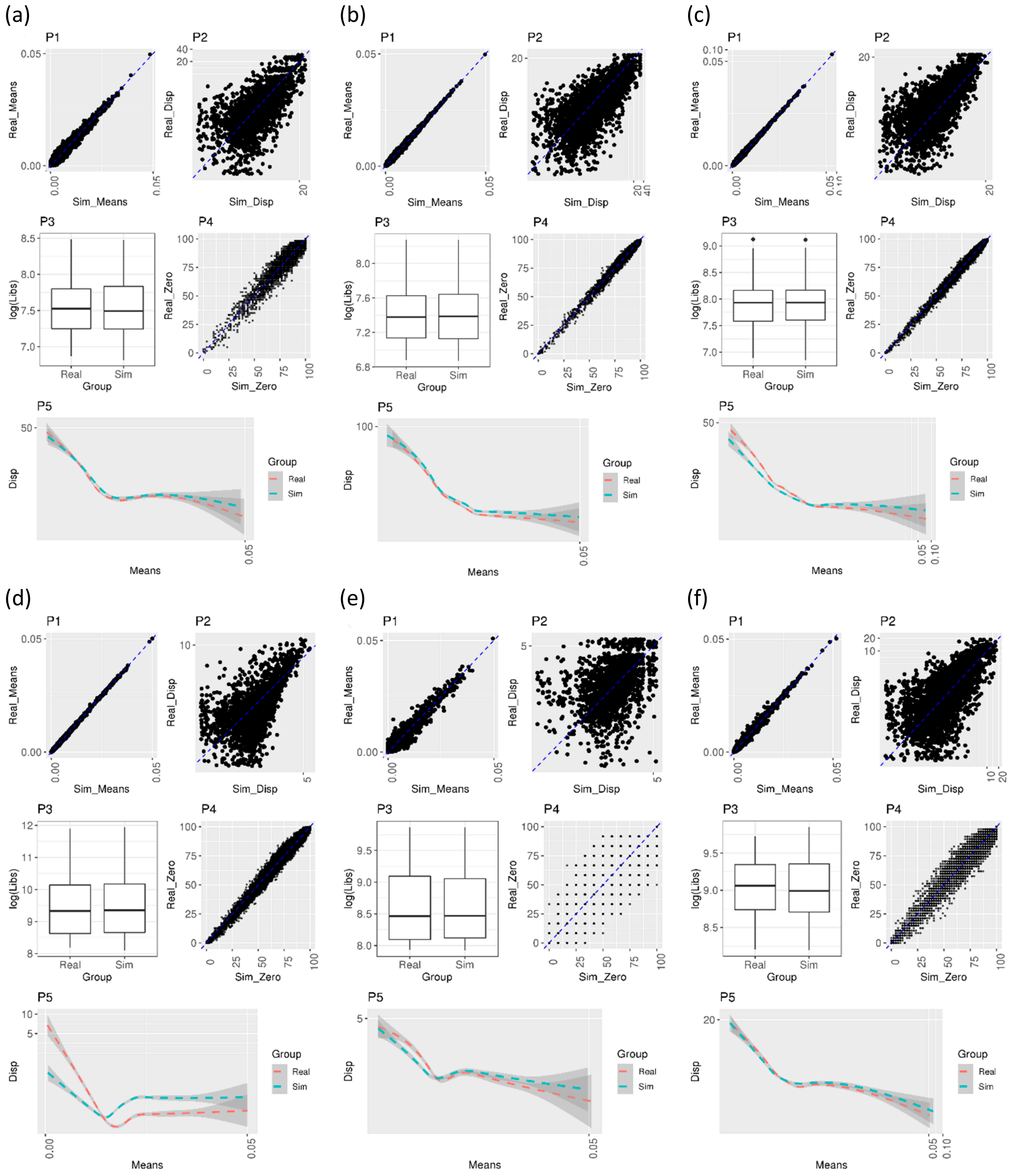

Figure S2. Diagnostic plots. (a) another reference group sample from EN-MIX cell type in Schirmer et al. (b)-(c) two reference group samples from Astrocytes cell type in Schirmer et al. (d) another reference group sample from AT1 cell type in Reyfman et al. (e)-(f) two reference group samples from SMC+Fibroblasts cell type in Reyfman et al.

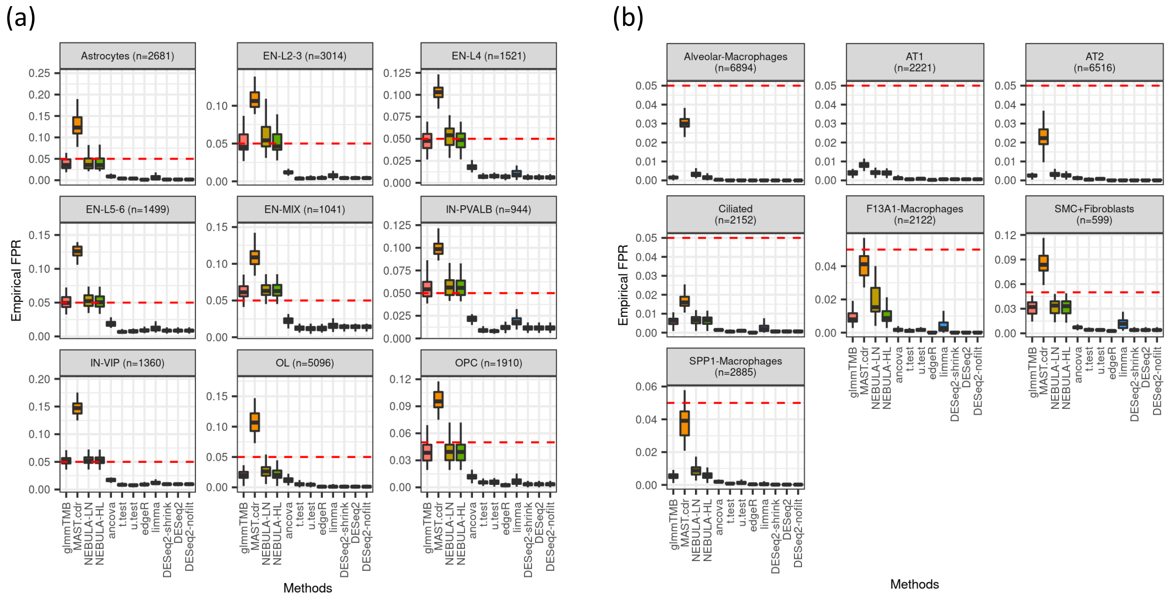

Figure S3. Distributions of observed false positive rate (FPR) given type-I error rate is 0.05 (red dotted line). (a) data was simulated based on Schirmer et al. and lowly-expressed genes were excluded by "or" filtering scheme (b) data was simulated based on Reyfman et al. and lowly-expressed genes were excluded by "or" filtering scheme.

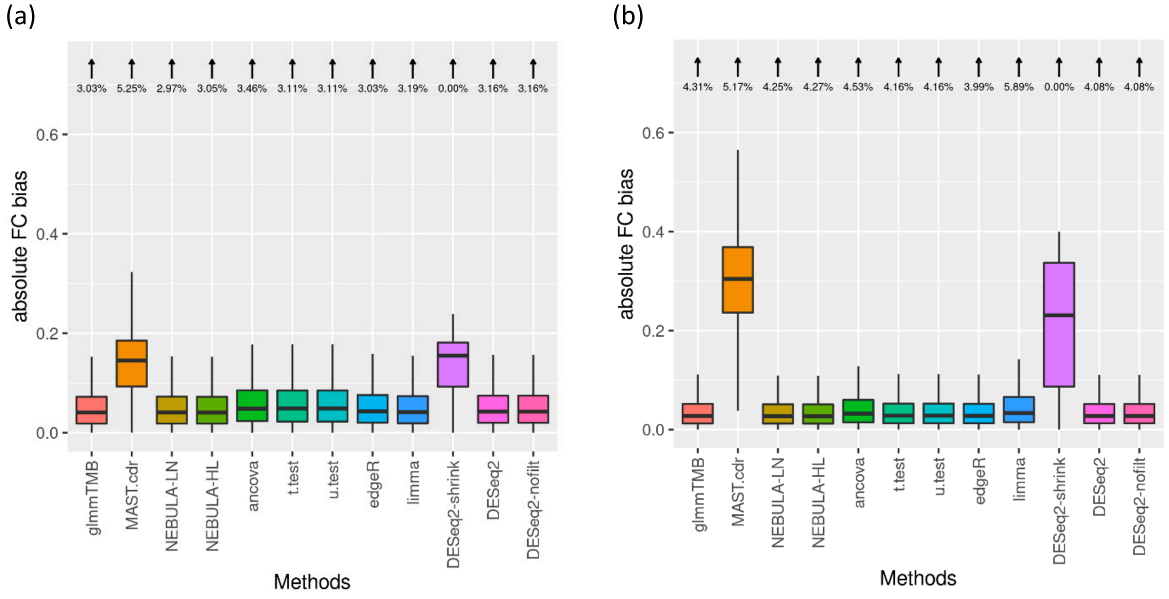

Figure S4. Boxplots of absolute FC bias (arrows denote the proportion of boxplot outliers). (a) EN-L4 cell type was simulated based on Schirmer et al. given FC=1.2 and "or" filtering scheme. (b) Alveolar-macrophages cell type was simulated based on Reyfman et al. given FC=1.4 and "or" filtering scheme. in

different simulation settings such as FC=1.5 and lowly-expressed genes were removed by “and” filtering scheme. (a) EN-L4 cells were simulated based on Schirmer et al. (b) Alveolar-macrophages cells were simulated based on Reyfman et al.

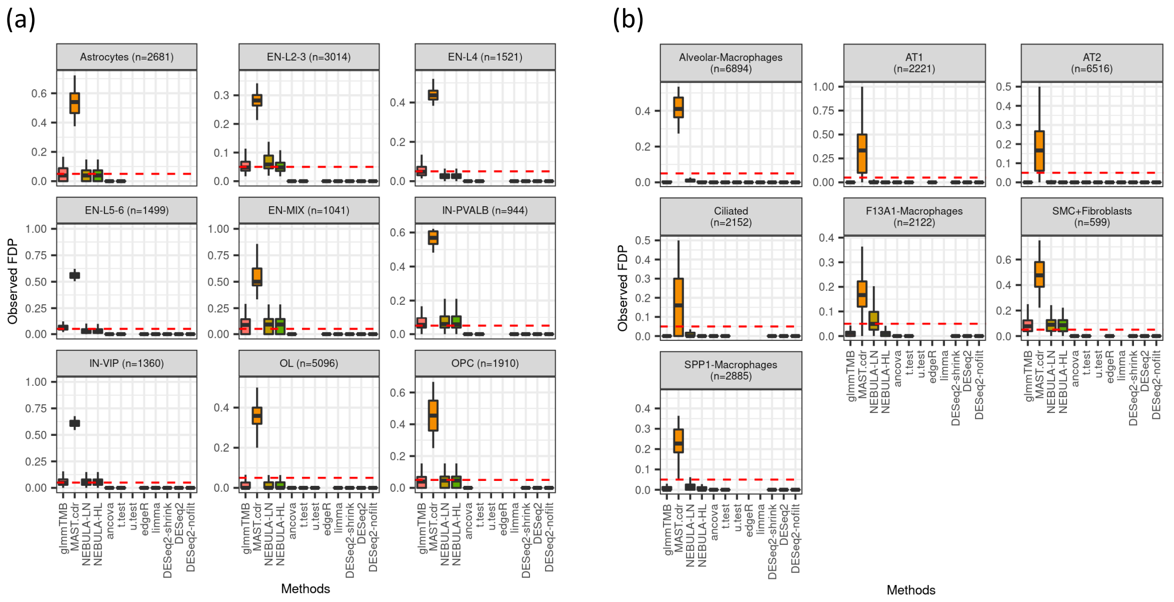

Figure S5. Distribution of observed false discovery proportion (FDP) given a fixed FDR is 0.05 (red dotted line). (a) data was simulated based on Schirmer et al. with FC=1.2 and lowly-expressed genes were excluded by “or” filtering scheme (b) data was simulated based on Reyfman et al. with FC=1.4 and lowly-expressed genes were excluded by “or” filtering scheme.

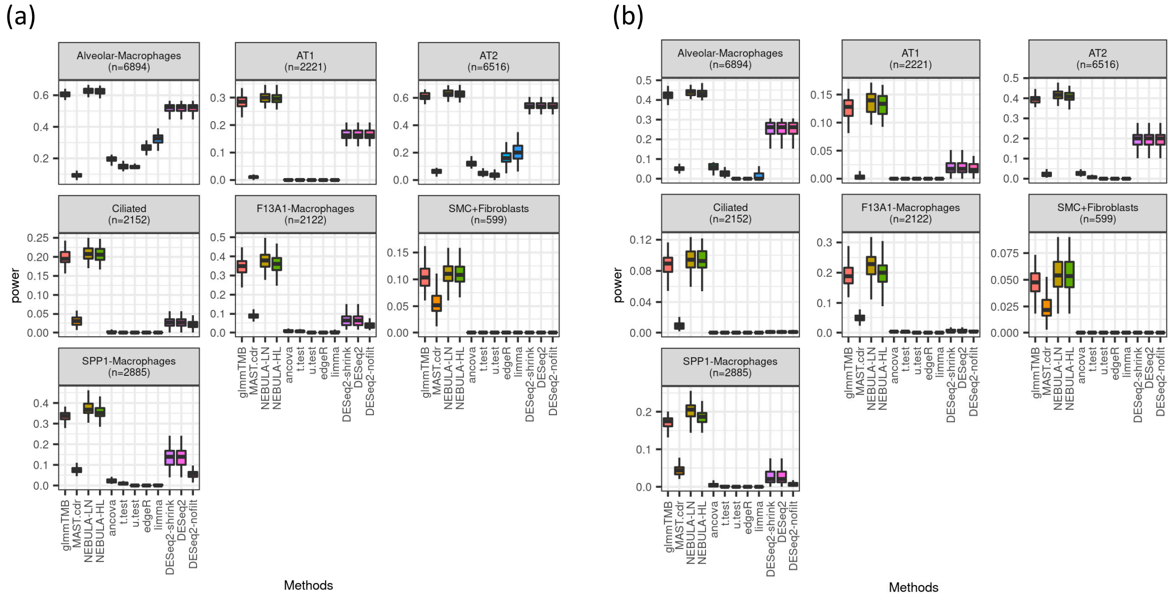

Figure S6. Distribution of power over 50 simulation data sets based on Reyfman et al. (a) FC is 1.5 and lowly-expressed genes were excluded by “and” filtering scheme. (b) FC is 1.4 and lowly-expressed genes were excluded by “or” filtering scheme.

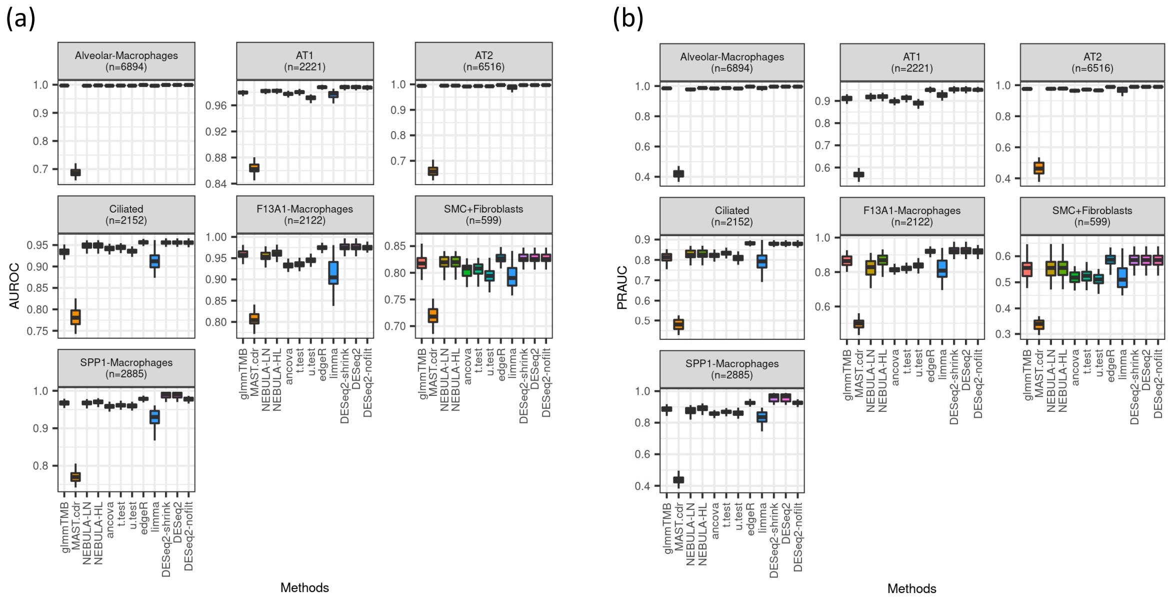

Figure S7. Distribution of AUROC and PRAUC over 50 simulation data sets based on Reyfman et al. given FC= 1.4 and lowly-expressed genes were excluded by “or” filtering scheme. (a) AUROC (b) PRAUC.

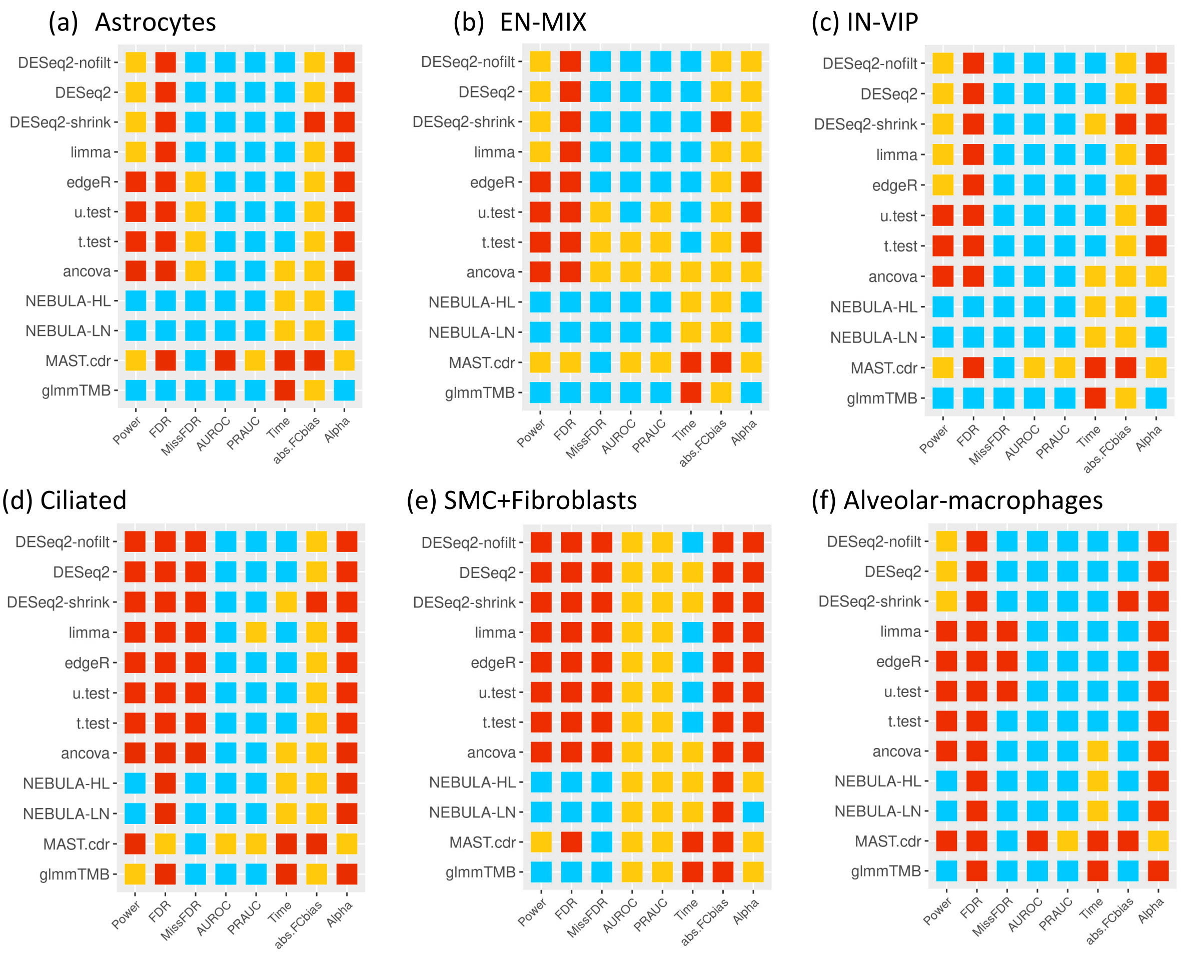

Figure S8. Heatmaps of 12 DE methods in a variety of overall performance metrics. (a-c) Astrocytes, EN-MIX, and IN-VIP cells given FC=1.5 and lowly expressed genes were excluded by “and” filtering scheme from Schirmer et al. (d-f) Ciliated, SMC+Fibroblasts, and Alveolar-macrophages cells given FC=1.4 and lowly-expressed genes were excluded by “or” filtering scheme from Reyfman et al.

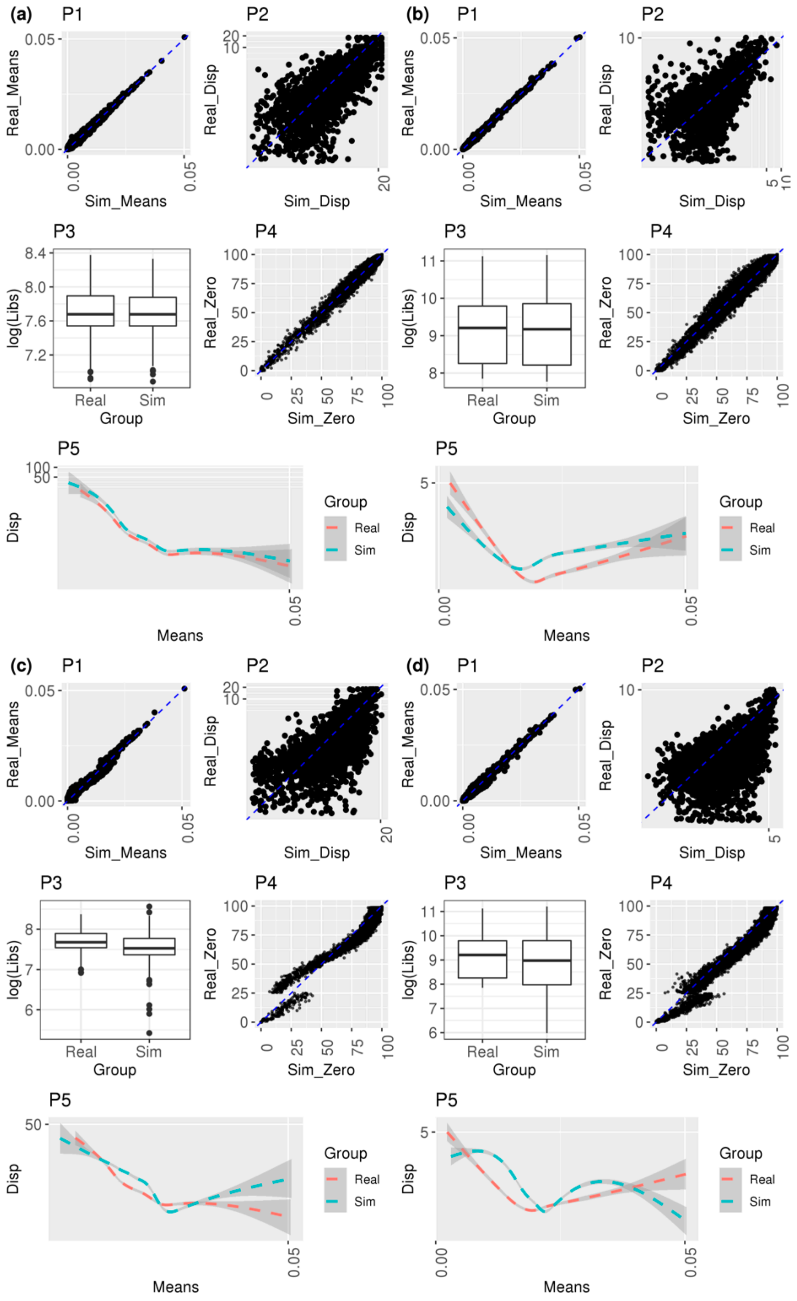

Figure S9. Diagnostic plots to compare simulation with real data using different simulation methods. (a) one control sample from EN-MIX cell-type in Schirmer et al. (b) one control sample from AT1 cell-type in Reyfman et al. (c) SPsimSeq simulation for control sample from EN-MIX cell-type. (d) SPsimSeq simulation for donor sample from AT1 cell type. P1: scatterplot of all gene means from real vs. simulated cell-level normalized counts, P2: scatterplot of filtered dispersions from real vs. simulated cell-level normalized counts, P3: boxplot of all library sizes from real vs. simulated cell-level normalized counts, P4: scatterplot of the proportion of zero counts from real vs. simulated cell-level normalized counts, and P5: Loess smoother with 95% confidence intervals of the relationship between the means and dispersions from real vs. simulated cell-level normalized counts.

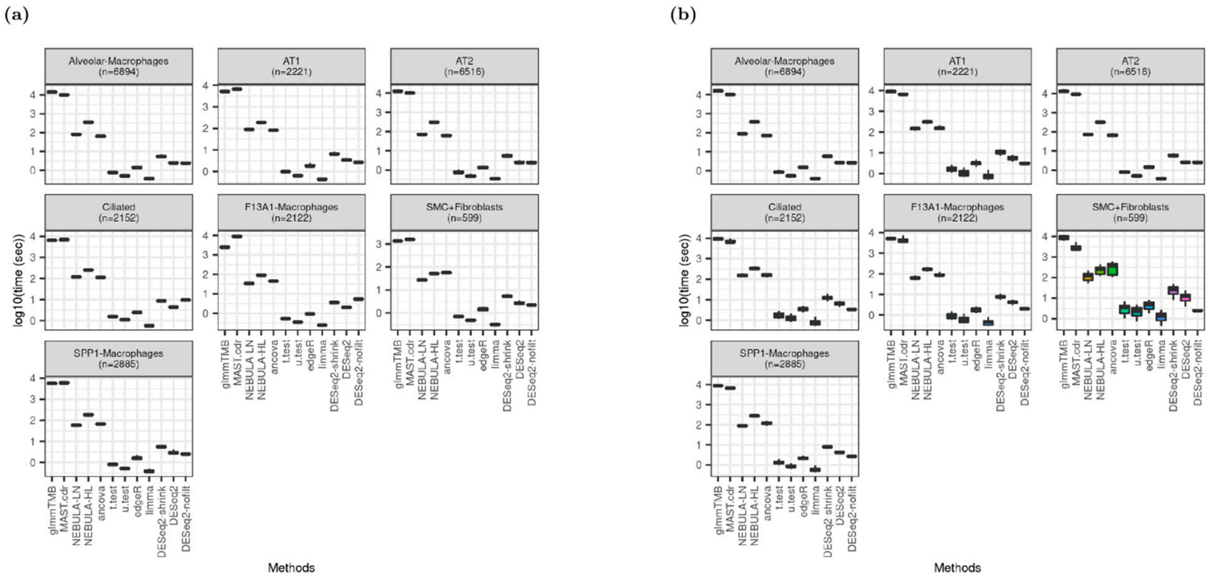

Figure S10. Boxplot of elapsed time in logarithmic scale at base 10 based on Reyfman et al. when FC=1.5. (a) using “and” filtering scheme. (b) using “or” filtering scheme.

Table S1. Enriched GO terms for cell type EN-L2-3 (Schirmer data)

Enriched GO terms for EN-L2-3	Schirmer paper	Ours (Y=All, N=None)
Translational Initiation	Y	Y
Chaperone-mediated Protein Assembly	Y	Y
Protein Targeting To Membrane	Y	Y
Nonsense-mediated Decay	Y	N
Viral Transcription	Y	N
Response To Unfolded Protein	Y	N
Protein Stabilization	Y	Y
Chaperone-dependent Protein Refolding	Y	Y
Ribosomal Subunit Assembly	Y	Y
Protein Localization To Telomere	Y	N
Apoptotic Mitochondrial Changes	N	Y
Oxidative Phosphorylation	N	Y
Cellular Response To Toxic Substance	N	Y
Neural Nucleus Development	N	Y (glmmTMB, NEBULA)
Response To Toxic Substance	N	Y
Response To Antibiotic	N	Y (glmmTMB, NEBULA)
Regulation Of Cell Death	N	Y

Table S2. Enriched GO terms for cell type EN-L4 (Schirmer data)

Enriched GO terms for EN-L4	Schirmer paper	Ours (Y=All, N=None)
Response To Unfolded Protein	Y	N
Chaperone-mediated Protein Assembly	Y	Y
Nervous System Development	Y	N
Chaperone-dependent Protein Refolding	Y	N
Protein Refolding	Y	N
Negative Regulation OF Cell Death	Y	N
Regulation Of Cellular Response To Heat	Y	Y
Multi-organism Process	Y	N
Apoptotic Mitochondrial Changes	N	Y
Oxidative Phosphorylation	N	Y
Cellular Response To Toxic Substance	N	Y
Response To Toxic Substance	N	Y
Response To Virus	N	Y (DESeq2, NEBULA)
Response To Oxidative Stress	N	Y
Response To Antibiotic	N	Y (glmmTMB, NEBULA)

Table S3. Enriched GO terms for cell type EN-L5-6 (Schirmer data)

Enriched GO terms for EN-L5-6	Schirmer paper	Ours (Y=All, N=None)
Chaperone-medicated Protein Assembly	Y	Y
Response To Unfolded Protein	Y	N
Translational Initiation	Y	Y
Chaperone-dependent Protein Refolding	Y	N
Protein Targeting To Membrane	Y	Y
Nonsense-mediated Decay	Y	N
Protein Refolding	Y	Y
Nervous System Development	Y	N
ATP Metabolic Process	Y	Y
Regulation Of Transport	Y	N
Protein Stabilization	N	Y
Cellular Response To Toxic Substance	N	Y
Response To Toxic Substance	N	Y
Oxidative Phosphorylation	N	Y

Table S4. Enriched GO terms for cell type Alveolar-macrophages (Reyfman data)

Enriched GO terms for Alveolar-macrophages	Reyfman Paper	Ours (Y=All, N=None)
Cell Matrix Adhesion	N	Y (NEBULA)
Cell Substrate Adhesion	N	Y
Cytoplasmic Translation	N	Y
Homotypic Cell Cell Adhesion	N	Y (DESeq2, NEBULA)
Inflammatory Response	N	Y (DESeq2, NEBULA)
Integrin Mediated Signaling Pathway	N	Y
Ion Transmembrane Transport	N	Y (DESeq2, NEBULA)
Immune System Process	Y	N
Extracellular Structure Organization	Y	N
Secretion	Y	N
Leukocyte Activation	Y	N
Developmental Process	Y	N

Table S5. Enriched GO terms for cell type AT2 (Reyfman data)

Enriched GO terms for AT2	Reyfman paper	Ours (Y=All, N=None)
Activation Of Immune Response	N	Y
Cell Activation Involved In Immune Response	N	Y (glmmTMB)
Cell Cell Adhesion	Y	Y
Cell Growth	N	Y (DESeq2, glmmTMB)
Cell Matrix Adhesion	N	Y (DESeq2, glmmTMB)
Cell Substrate Adhesion	N	Y
Immune Effector Process	N	Y
Inflammatory Response	N	Y
Innate Immune Response	N	Y
Leukocyte Cell Cell Adhesion	N	Y
Leukocyte Mediated Immunity	N	Y
Response To Wounding	N	Y
Supramolecular Fiber Organization	N	Y
Wound Healing	N	Y
Developmental Process	Y	N
Response To Stimulus	Y	N
Granulocyte Activation	Y	N
Regulation Of Programmed Cell Death	Y	N
Interferon-Gamma-Mediated Signaling Pathway	Y	N
